# Microwave-Assisted Synthesis of Glycoconjugates by Transgalactosylation with Recombinant Thermostable β-Glycosidase from *Pyrococcus* †

**DOI:** 10.3390/ijms17020210

**Published:** 2016-02-04

**Authors:** Manja Henze, Dorothee Merker, Lothar Elling

**Affiliations:** Laboratory for Biomaterials, Institute of Biotechnology and Helmholtz-Institute for Biomedical Engineering, RWTH Aachen University, 52074 Aachen, Germany; m.henze@biotec.rwth-aachen.de (M.H.); d.merker@biotec.rwth-aachen.de (D.M.)

**Keywords:** biocatalysis, glycosidase, carbohydrates, glycoconjugates, microwave irradiation, transgalactosylation, *Pyrococcus*

## Abstract

The potential of the hyperthermophilic β-glycosidase from *Pyrococcus woesei* (DSM 3773) for the synthesis of glycosides under microwave irradiation (MWI) at low temperatures was investigated. Transgalactosylation reactions with β-*N*-acetyl-d-glucosamine as acceptor substrate (GlcNAc-linker-*t*Boc) under thermal heating (TH, 85 °C) and under MWI at 100 and 300 W resulted in the formation of (Galβ(1,4)GlcNAc-linker-*t*Boc) as the main product in all reactions. Most importantly, MWI at temperatures far below the temperature optimum of the hyperthermophilic glycosidase led to higher product yields with only minor amounts of side products β(1,6-linked disaccharide and trisaccharides). At high acceptor concentrations (50 mM), transgalactosylation reactions under MWI at 300 W gave similar product yields when compared to TH at 85 °C. In summary, we demonstrate that MWI is useful as a novel experimental set-up for the synthesis of defined galacto-oligosaccharides. In conclusion, glycosylation reactions under MWI at low temperatures have the potential as a general strategy for regioselective glycosylation reactions of hyperthermophilic glycosidases using heat-labile acceptor or donor substrates.

## 1. Introduction

Glycosidases are widely used among the well-established enzymatic synthesis strategies of glycoconjugates [1]. The main drawback in thermodynamically and kinetically driven synthesis reactions is the relatively low product yield due to secondary product hydrolysis. Reaction engineering strategies to lower product hydrolysis includes reaction conditions at high substrate concentrations with addition of organic solvents or ionic liquids, as well as synthesis in frozen solutions [2,3]. In terms of enzyme stability, numerous glycosidases from thermophilic microorganisms were favorably utilized [4]. However, their optimum activity at temperatures between 80 and 110 °C may be of disadvantage for optimizing transglycosylation reactions in aqueous buffer solutions using heat-labile substrates or products. However, performance of synthesis reactions at ambient temperature may not be effective with glycosidases from extreme thermophiles and affords sophisticated reaction engineering.

The beneficial use of microwave irradiation (MWI) has been demonstrated for synthetic chemical reactions leading to greener protocols resulting in reduction of waste and reaction times [5,6,7,8]. However, there is still an ongoing debate whether the observed effects can be attributed to thermal dielectric microwave heating or explained by non-thermal specific microwave effects [9].

In biocatalysis, MWI has been used in reactions employing hydrolases (lipases and glycosidases), isomerases, and decarboxylases with beneficial effects for activity and selectivity [10,11,12,13,14,15,16]. With mesophilic enzymes, the lack of thermal stability under microwave heating has been overcome by enzyme immobilization or the use of ionic liquids [16,17,18,19]. We recently demonstrated for two mesophilic β-galactosidases that product hydrolysis is avoided in transglycosylation reactions due to controlled enzyme inactivation by MWI [20,21]. Thermophilic enzymes appear to be more suitable for reactions under MWI due to their higher thermostablity. However, they have also been found to be inactivated by MWI [22,23,24] at temperatures where they were highly stable under conventional thermal heating. In contrast, CelB from *Pyrococcus furiosus* showed hydrolytic activity under MWI at ambient temperatures in comparison to conventional thermal heating being not active [12]. To the best of our knowledge, the concept of microwave-assisted transglycosylation reaction using non-immobilized hyperthermophilic biocatalysts has not been evaluated so far.

In this study, we focused on *Pyrococcus woesei* β-d-galactosidase (GenBank accession number AF043283.1) clustered in glycoside hydrolase 1 (GH-1) family with a retaining catalytic mechanism. The hyperthermophilic enzyme has a high sequence identity (99.8%) to the β-galactosidase of *Pyrococcus furiosus* [25] and has been recombinantly produced in *E. coli* expression strains and characterized for its hydrolytic activity [25,26,27,28]. Directed evolution of the synthetic gene (GenBank accession number EF090269) switched the β-galactosidase activity to β-glucuronidase activity by exchange of seven key amino acid residues [29]. The identical gene sequence was characterized as β-d-mannosidase from another species of *Pyrococcus*, *Pyrococcus furiosus* [30] (Figure S1, supplementary materials). This is not surprising due to the fact that both thermophilic archaea exhibit a high evolutionary relationship [31] and duplicate gene copies can be translated into proteins, which display hydrolytic activity for stereoisomers [30]. However, this enzyme originating from shallow marine volcanic vents [31] facilitates universal biotechnological applications supported by the choice of donor substrates at very high temperatures. Optimal hydrolytic enzymatic activities were reported up to 100 °C [27,30]. 

**Scheme 1:**
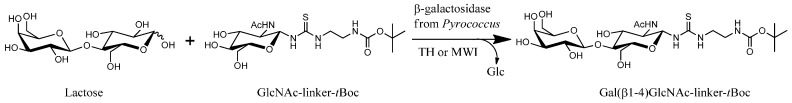
Transgalactosylation reactions of β-glycosidase from *Pyrococcus woesei* DSM 3773 for the production of the main disaccharide product under conventional thermal heating (TH) or microwave irradiation (MWI).

We here report for the first time on microwave-assisted transglycosylation reactions employing a hyperthermophilic glycosidase from *Pyrococcus woesei* DSM 3773 strain (DSM, Deutsche Sammlung von Mikroorganismen, German Collection of Microorganism) at temperatures far below its temperature optimum. In this work, the transgalactosylation activity was selected by applying high concentrations of lactose as donor and GlcNAc-linker-*t*Boc as acceptor substrate (Scheme 1). Fixed microwave power intensities up to 300 W at temperatures between 12 and 30 °C enhance the hydrolytic and synthetic reaction performances compared with those temperatures under conventional thermal heating conditions. Our results present the beneficial application of MWI for the synthesis of glycoconjugates with a hyperthermophilic biocatalyst far below its temperature optimum. This technological strategy should also broaden the scope of reactions using hydrolytically inactive glycosidases (glycosynthases).

## 2. Results and Discussion

### 2.1. Production and Characterization of Recombinant β-Glycosidase

The gene encoding β-galactosidase from *Pyrococcus woesei* strain DSM 3773 (GenBank accession No. AF043283.1) was cloned from genomic DNA and inserted into the pET-Duet™-1 vector resulting in a fusion to an N-terminal His_6_-tag. Dabrowski *et al.* reported dissimilarity of two triplets in β-galactoside hydrolase genes between the hyperthermophilic strains *Pyrococcus woesei* and *Pyrococcus furiosus*, whereby only one nucleotide variation resulted in an amino acid replacement from isoleucine (*P. furiosus*) to threonine (*P. woesei*) at site 436 [25]. Sequence analysis of the cloned gene revealed changes in two nucleotides resulting in only one exchange of amino acid at position 436 (from threonine to isoleucine) (Figures S1 and S2), which is characteristic of *P. furiosus* β-galactosidase gene (GenBank accession number E08095.1) [25] and the *bmnA* gene encoding a β-mannosidase from *P. furiosus* (GenBank accession no. AAC44387.1) [30] (Figure S1). Due to different codon usage of the thermophilic archaea and mesophilic bacteria the *E. coli* expression strain Rosetta2™(DE3)pLysS was used for efficient enzyme production. Cultivation of *E. coli* Rosetta2™(DE3)pLysS gave the averaged cell mass of 14.5 g/L. Homogeneous enzyme preparations were obtained by the combination of double heat treatment at 75 and 85 °C and immobilized metal ion chromatography (IMAC) (Figures S3 and S4) as previously shown by Wanarska *et al.* [28]. The purification protocol of β-glycosidase from *Pyrococcus* produced in *E. coli* Rosetta2™(DE3)pLysS (5 g cell mass) yielded 600 U with a volumetric activity of 76 U/mL and a specific activity of 107 U/mg. A subsequent buffer exchange resulted in a homogeneous enzyme preparation with a specific activity of 58 U/mg.

We first investigated the substrate spectrum for the hydrolysis of various nitrophenyl (NP)-glycosides. Kinetic data between 0.8 and 2.9 mM were already described [27,30]. We therefore tested substrate concentrations between 15 and 30 mM for optimal activity measurements (Table 1). Surprisingly, the enzyme showed highest activity for *p*NP-β-d-Glc and considerable activities for *p*NP-β-d-Gal, *p*NP-β-d-Man as well as *p*NP-β-d-Xyl. In conclusion, the enzyme depicts a promiscuous substrate specificity which may also be exploited for transglycosylation reactions. Further characterization revealed highest β-galactosidase activity at pH 5.5 as previously reported [26]. The optimum enzyme activity was measured at a temperature of 85 °C for *p*NP-β-d-Gal hydrolysis (Figure S5).

**Table 1:** Substrate specificity of recombinant β-glycosidase from *Pyrococcus woesei* strain DSM 3773 for the hydrolysis of aryl glycosides at 85 °C in 25 mM citrate-phosphate buffer, pH 5.5.

Aryl Glycosides	Relative Activity (%) ^[c]^
*p*-Nitrophenyl-β-d-glucopyranoside (*p*NP-β-Glc) ^[a]^	100
*p*-Nitrophenyl-β-d-galactopyranoside (*p*NP-β-Gal) ^[a]^	89
*o*-Nitrophenyl-β-d-galactopyranoside (*o*NP-β-Gal) ^[a]^	69
*p*-Nitrophenyl-β-d-mannopyranoside (*p*NP-β-Man) ^[b]^	39
*p*-Nitrophenyl-β-d-xylopyranoside (*p*NP-β-Xyl) ^[a]^	15

^[a]^ 30 mM; ^[b]^ 15 mM aryl substrates; ^[c]^ 100% corresponds to 21 U/mL.

We investigated the hydrolytic and transglycosylation activity in the presence of β4-linked disaccharides such as cellobiose, lactose, and lactulose. In all cases, transglycosylation products were observed yielding also galacto-oligosaccharides in the case of lactose as substrate (Figure S6). The synthesis of lactulose and galactosyl-oligosaccharides from lactose and d-fructose was demonstrated by Grubiak *et al.* [32]. We further studied the regiospecificity of the recombinant β-glycosidase from *Pyrococcus* with previously synthesized Galβ(1,3/4/6)GlcNAc-linker-*t*Boc regioisomers [33,34,35]. The recombinant enzyme shows higher preference for the hydrolysis of β(1,4)- than for β(1,6)-linked galactosides. However, Galβ(1,3)GlcNAc-linker-*t*Boc was also cleaved after prolonged incubation time (Figures S7–S9).

### 2.2. Hydrolytic Activity of Recombinant β-Glycosidase from Pyrococcus under MWI

We aimed to demonstrate that hyperthermophilic enzymes are active under microwave irradiation far below their optimum temperature. Indeed, in comparison to thermal heating (TH) at 30 °C a three-fold higher hydrolytic activity of recombinant β-glycosidase from *Pyrococcus* is observed under MWI at 300 W power input (Table 2). Notably, hydrolytic activity can be tuned by power input under MWI reaching five-fold higher activity by variation from 100 to 300 W. The hydrolytic activity at the corresponding temperatures (<30 °C) under TH is far below those measured at MWI (Table 2; Figure S5). Stirring the reaction mixture did not influence the hydrolytic activity (Table S1). We concluded that hotspots of heat induced by MWI do not play a role. As a consequence, all further experiments were performed without stirring.

**Table 2:** Hydrolytic activity of recombinant β-glycosidase from *Pyrococcus woesei* strain DSM 3773 under fixed microwave irradiation energy (MWI) in comparison to conventional thermal heating (TH). Release of *p*-nitrophenol (*p*NP) from 30 mM *p*NPGal in 25 mM citrate-phosphate buffer pH 5.5 was continuously monitored. For MWI experiments, the temperature range measured by the fiber optic sensor at the given energy input is depicted–100% corresponds to 19 U/mL.

Reaction Condition	Relative Activity (%)	Temperature (°C)
TH 85 °C	100.0	85 °C
TH 30 °C	6.2 ± 3.0	30 °C
MWI 100 W	4.3 ± 1.4	8–12 °C
MWI 200 W	10.3 ± 2.9	10–14 °C
MWI 300 W	21.3 ± 2.9	20–32 °C

**Figure 1:**
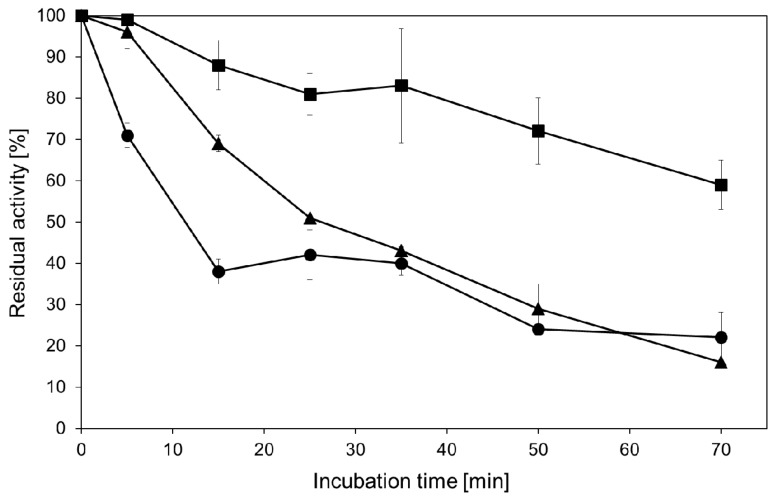
Stability of hyperthermophilic β-glycosidase from *Pyrococcus* under conventional thermal heating at 85 °C and MWI at 100 and 300 W, respectively. Residual β-galactosidase activity (*p*NP-β-Gal) was assayed after incubation in a conventional heating block (■) or under microwave irradiation at 100 W (▲) or 300 W (●). Assayed enzyme activity under standard condition was used as a reference (100% corresponds to 17 U/mL).

In this respect, our results confirm the previous study with the hyperthermophilic β-glucosidase (CelB) from *P. furiosus*. Here, a microwave-inducible effect was observed by an increased hydrolytic activity for a rising electric field in a temperature range from −20 to 40 °C [12]. We further investigated the enzyme stability under MWI. Figure 1 demonstrates that MWI affects the stability of β-glycosidase from *Pyrococcus* with a higher rate of inactivation for 100 and 300 W, respectively, when compared to conventional thermal heating at 85 °C. After a pre-incubation time of 70 min, 25% residual hydrolytic activity is found. The impact of power input levels off after longer incubation time. It should be noted that the addition of sucrose up to 1 M and the use of the ionic liquid [BMIM] [PF6] stabilizes the enzyme activity in experiments performed at 85 °C under conventional thermal heating conditions (Figure S10). The decreased enzyme stability under MWI may not be transferred to reaction conditions for transgalactosylation reactions as performed below since high donor concentrations (600 mM lactose) may also stabilize the enzyme under these conditions.

### 2.3. Transglycosylation Reactions with Recombinant β-Glycosidase from Pyrococcus under Thermal Heating

**Figure 2:**
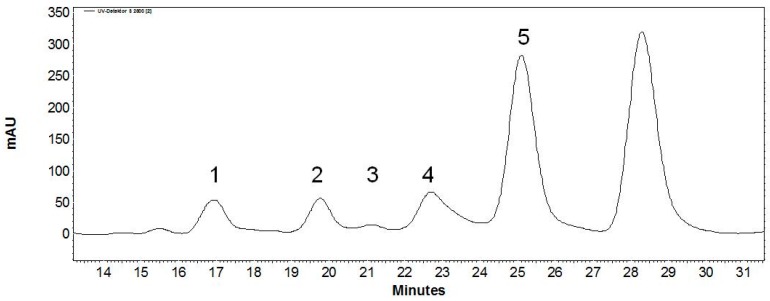
Semi-preparative Reversed-Phase (RP)-HPLC separation of pooled product fractions from transgalactosylation reactions of recombinant β-glycosidase from *Pyrococcus*. 10 mM β-d-GlcNAc-linker-*t*Boc, 600 mM lactose and 12 U/mL enzyme solution were incubated for 30 min at 85 °C under conventional thermal heating. Single product peaks were collected and analyzed by analytical RP-HPLC, enzymatic digestion, ESI-MS measurements and NMR spectroscopy (see supplementary materials).

The acceptor β-d-GlcNAc-linker-*t*Boc and lactose as donor substrate were chosen to compare the product formation for transgalactosylation reactions under thermal heating (85 °C) and MWI. Kinetic analysis at standard conditions (thermal heating 85 °C) revealed Michaelis–Menten kinetics with an apparent *K*_m_ value of 15.6 mM and apparent *v*_max_ of 76 U/mg for the variable substrate β-d-GlcNAc-linker-*t*Boc (Figure S11). Based on our data that *Pyrococcus* β-galactosidase is able to cleave β(1,3/4/6)-glycosidic linkages, we expected a mixture of disaccharide regioisomers and oligomers. Therefore, we first analyzed the products for a transgalactosylation reaction under thermal heating (85 °C) conditions (Figure 2). The product fractions were isolated and analyzed by High Performance Liquid Chromatography with Electrospray Ionization Mass Spectrometry (HPLC/ESI-MS) (Figures S12 and S13).

The main product (peak 5, 41.5% relative amount) is a disaccharide (Table 3). Further analysis of its identity was performed by comparing the retention time with regioisomers of corresponding disaccharide standards (Figure S14) as well as cleavage analysis with specific commercial β-galactosidases (Figure S15). Summarizing the results from these experiments, we conclude that Galβ(1,4)GlcNAc-linker-*t*Boc is formed as the main product under the applied reaction conditions. This was finally confirmed by NMR-analysis (Figure S16). The product mixture comprises one further disaccharide with 3.3% (peak 2, Figure 2 and Table 3), probably the β(1,6)-linked product, and three trisaccharides in an overall relative amount of 9.9%, all eluting at different retention times (Figure 2 and Table 3).

**Table 3:** Observed molecular mass of isolated product fractions analyzed on analytical HPLC. Transgalactosylation reaction (10 mM β-d-GlcNAc-linker-*t*Boc, 600 mM lactose and 12 U/mL enzyme solution) under thermal heating for 30 min at 85 °C.

Peak	Retention Time (min)	Relative Peak Area (%)	*m*/*z* Found [M − H]^−^	*m*/*z* Calculated [M]	Product
1	9.6	3.8	745.2	746.7	Gal-Gal-GlcNAc-linker-*t*Boc
2	11.3	3.3	583.1	584.6	Gal-GlcNAc-linker-*t*Boc
3	12.3	0.3	745.2	746.7	Gal-Gal-GlcNAc-linker-*t*Boc
4	13.4	5.8	745.2	746.7	Gal-Gal-GlcNAc-linker-*t*Boc
5	14.9	41.5	583.2	584.6	Gal(β1,4)GlcNAc-linker-*t*Boc

### 2.4. Transglycosylation Reactions with Recombinant β-Glycosidase from Pyrococcus under MWI

**Figure 3:**
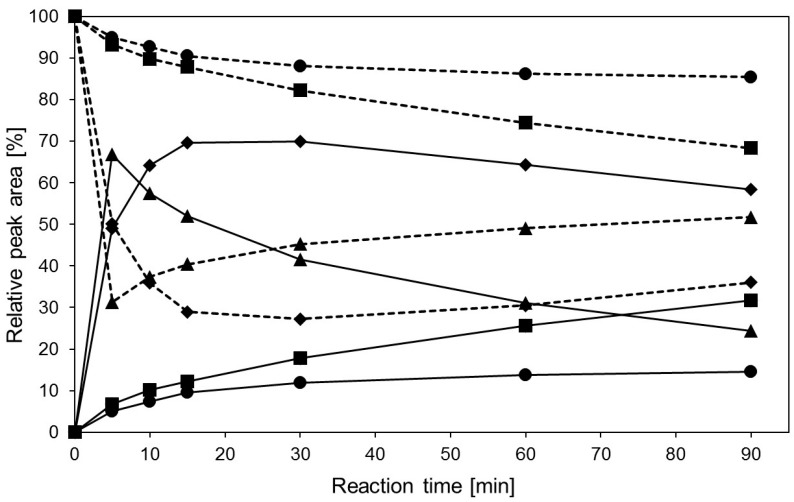
Transgalactosylation reaction with hyperthermophilic β-glycosidase from *Pyrococcus* under MWI and TH starting from 10 mM GlcNAc-linker-*t*Boc. Analytical HPLC were used for monitoring acceptor substrate (GlcNAc-linker-*t*Boc, dashed lines) and the main product (Galβ(1,4)GlcNAc-linker-*t*Boc, solid lines) at 254 nm. Experimental conditions: 100 W MWI (■) with an average temperature of ~20 °C, 300 W MWI (♦) with an average temperature of ~30 °C, 85 °C TH (▲) and 20 °C TH (●). The reaction mixture (1 mL) contained 12 U enzyme, 10 mM GlcNAc-linker-*t*Boc and 600 mM lactose in 25 mM citrate-phosphate buffer pH 5.5. The measured data with standard deviations are listed in Table S4.

The product formation of transgalactosylation reactions under MWI (100 and 300 W) and conventional thermal heating (TH, 85 and 20 °C) was compared. Figure 3 depicts the formation of the main product Galβ(1,4)GlcNAc-linker-*t*Boc over the reaction time. The yield of 70% was slightly higher under MWI (300 W, 30 °C) than under TH at 85 °C (67%). However, time points to reach highest yields were at 5 and 15 min, respectively. The product composition under TH at 5 min consists of a disaccharide with 1% and 0.9% trisaccharides (Table S2). Most importantly, product hydrolysis started immediately after 5 min under TH (85 °C) whereas a stable product concentration was obtained up to 30 min reaction time under MWI (300 W) (residual hydrolytic activity 40%, see Figure 1). We conclude that applying MWI (300 W) at a reaction temperature of 30 °C far below the temperature optimum of the hyperthermophilic glycosidase leads to a similar product yield with delayed product hydrolysis. In addition, the formation of side products is also delayed under MWI reaching lower yields with an overall amount of 4.8% after 60 min compared to 19.3% synthesized under conventional thermal heating condition (Figure S17A,D, Table S2). In conclusion, we rate MWI at 300 W as favorable for the transgalactosylation reaction of hyperthermophilic β-glycosidase from *Pyrococcus*. The delay in product hydrolysis and formation of further products may be rationalized by a lower overall enzyme activity at 30 °C (Figure S5), however, reaching the same product yield suggests that MWI may tune enzyme activity. This conclusion is further supported by a higher product yield under MWI (100 W) at 20 °C when compared to TH at 20 °C (Figure 3). In both cases, only the main product is formed (Figure S17B,C). All products were stable under MWI at 300 W (Figure S18 and Table S3). The main product synthesized under MWI was verified by NMR-analysis (Figure S16).

**Figure 4:**
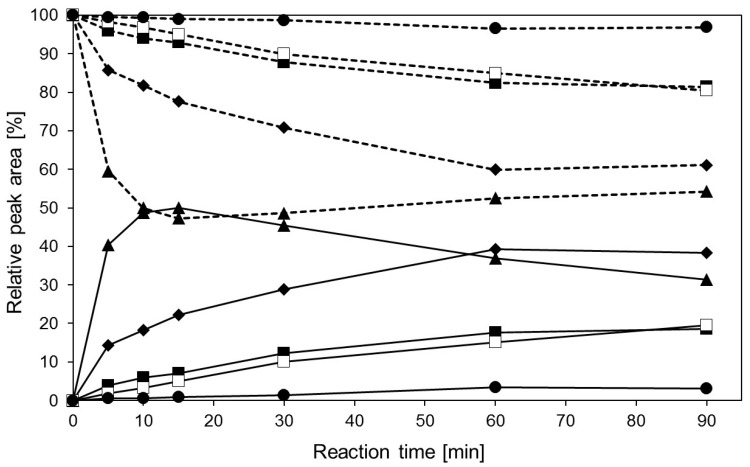
Comparison of transgalactosylation reactions with recombinant β-glycosidase from *Pyrococcus* under MWI and TH starting from 50 mM GlcNAc-linker-*t*Boc. Analytical HPLC were used for detection of reactant (GlcNAc-linker-*t*Boc, dashed lines) and main product (Galβ(1,4)GlcNAc-linker-*t*Boc, solid lines) at 254 nm. Experimental conditions: 100 W MWI (■) with an average temperature of ~12 °C, 300 W MWI (♦) with an average temperature of ~25 °C, 85 °C TH (▲), 30 °C TH (□), and 12 °C TH (●). 1 mL reaction mixture contained 12 U/mL enzyme solution and 600 mM lactose in 25 mM citrate-phosphate buffer pH 5.5. The measured data with standard deviations are listed in Table S6.

The performance of the transgalactosylation reactions with a five-fold higher acceptor substrate concentration (50 mM), and the same enzyme amount resulted in product yields of 50% after 15 min under thermal heating at 85 °C (side products 2.3%, Table S5) and 40% after 60 min under 300 W MWI (side products 0.7%, Table S5), respectively (Figure 4). The higher product yield under conventional thermal heating conditions may reflect the higher enzyme stability and overall effective enzyme activity during synthesis. Surprisingly, yet a similar product concentration is obtained under MWI at 300 W after 60 min at a remarkably lower relative hydrolytic activity of only 20% (Table 2). We may conclude that, with respect to fast product formation, using the same enzyme concentrations MWI is not favorable. However, with MWI only <1%, Galβ(1,6)GlcNAc-linker-*t*Boc (peak 2 in Figure 2) is formed compared to overall side products of 10.7% produced under TH with approximately the same yield of the β4-linked disaccharide (Figure S19A,E, Table S5). In contrast, with respect to reaction temperatures far below the temperature optimum of the hyperthermophilic enzyme, MWI leads to 15% higher product yields with Galβ(1,4)GlcNAc-linker-*t*Boc as the main or sole product (Figure S19). This holds for MWI at 300 W (25 °C) and 100 W (12 °C) when compared to the reaction at temperatures (30 and 12 °C) under thermal heating (Figure 4).

**Figure 5:**
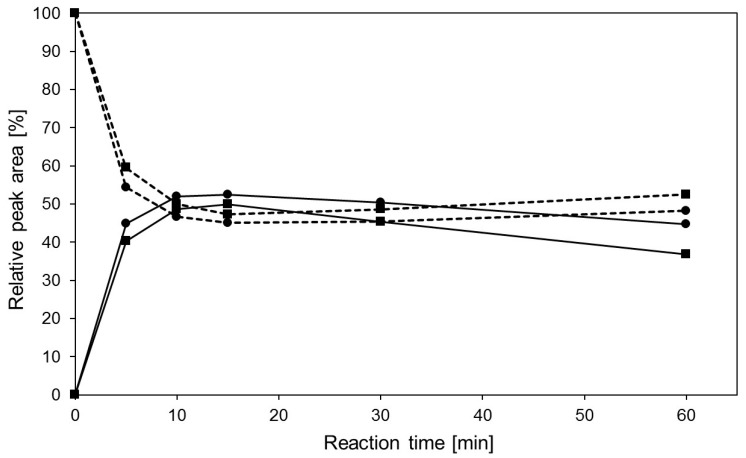
Transgalactosylation recombinant β-glycosidase from *Pyrococcus* under thermal heating at 85 °C (■) with 12 U/mL in comparison with transgalactosylation under MWI at 300 W (●) (average temperature of ~35 °C) including a five times higher enzyme activity (60 U/mL). Samples were separated on analytical HPLC at 254 nm: GlcNAc-linker-*t*Boc (dashed lines) and main product (Gal(β1,4)GlcNAc-linker-*t*Boc, solid lines). In addition, 1 mL reaction mixture contained 50 mM GlcNAc-linker-*t*Boc and 600 mM lactose in 25 mM citrate-phosphate buffer pH 5.5. The measured data with standard deviations are listed in Table S8.

In view of the fact that the production of the enzyme is not a limiting factor, catalytic reactions with the identical nominal enzyme activity were performed (Figure 5). We demonstrate here that the same reaction performance is obtained under MWI. A similar product yield of 50% (25 mM) can be achieved during the same period compared to conventional thermal heating at the optimum temperature. The synthesis of different linked glycoconjugates is slightly reduced under MWI after 60 min (Figure S20, Table S7). This result supports the beneficial use of the specific non-thermal microwave effect. Hence, our approach can be adopted as general synthetic strategy for regioselective glycosylation reactions with heat-labile acceptor or donor substrates catalyzed at low temperatures under MWI with maximum enzyme activity.

## 3. Materials and Methods

### 3.1. Gene Cloning

Genomic DNA from *Pyrococcus woesei* DSM 3773 (Leibniz Institute DSMZ-German Collection of Microorganisms and Cell Cultures, Braunschweig, Germany) was used to amplify the β-galactosidase gene (GenBank: AF043283.1). KOD Hot Start DNA-Polymerase Kit (Novagen/Merck, Darmstadt, Germany) was chosen for PCR reactions (30 μL sterile water, 5 μL 10× buffer, 4 μL 25 mM MgSO_4_, 5 μL 2 mM dNTP, 1.5 μL 10 µM forward primer, 1.5 μL 10 μM reverse primer, 1 μL DMSO, 1 μL DSM 3773 1 to 10 dilution, 1 μL of 1 U/μL KOD Polymerase) in the thermocyler (Master Cycler Gradient, Eppendorf, Hamburg, Germany) with the succeeding steps: initial denaturation for 2 min at 95 °C, denaturation for 20 s at 95 °C, annealing for 10 s at 70 °C, elongation for 30 s at 70 °C, repetition of 25 cycles with final elongation for 10 min at 70 °C and cooling at 4 °C. For the following sticky-end ligation into pET-Duet™-1 vector (Novagen, Darmstadt, Germany) the designed two primers for amplification contain the restriction sites for *SacI* or *SalI*: forward primer 5’-CGAGCTCGATGTTCCCTGAAAAGTTCCTTTGGGGTG-3’ (*SacI* site, restriction recognition site is underlined) and reverse primer 5’-ACGCGTCGACTCATCCCCTCAGCAATTCCTCTTCAATC-3’ (*SalI* site, restriction recognition site is underlined). PCR products were purified by *Nucleo Spin Extract II Kit* (Macherey und Nagel, Düren, Germany). To clone the galactosidase gene into the pET-Duet™-1 expression vector, selected for insertion of an N-terminal His-tag, both DNA-sequences were cut with *FastDigest SalI* and *FastDigest SalI* (Fermentas, St. Leon-Rot, Germany) at 37 °C following the suggested reaction conditions. The two constructs were ligated with T4 Ligase (Fermentas, St. Leon-Rot, Germany). The correctness of the pET-Duet™-1 His_6_βGal was confirmed by sequencing (StarSEQ, Germany). The plasmid was transformed by the heat shock method (1 μL ligation sample mixed with 25 μL cells on ice, 4 °C for 3 min, 42 °C for 30 s, 4 °C for 2 min) into *E. coli* NovaBlue Singles™ for multiplication or into *E. coli* Rosetta2(DE3)pLysS (Novagen, Darmstadt, Germany) for overexpression of the recombinant protein.

### 3.2. Production of Recombinant Glycosidase

A pre-culture of 20 mL LB medium in a 100 mL Erlenmeyer flask (all media were supplemented with corresponding antibiotics: 100 µg/mL ampicillin, 34 μg/mL chloramphenicol) was inoculated with transformants and agitated overnight (37 °C, 120 rpm). The main culture with 1 L TB media in a 5 L Erlenmeyer flask was inoculated with 1% (*v*/*v*) of pre-culture and incubated at 37 °C with shaking at 80 rpm. Production of the recombinant protein was induced by 100 mM isopropyl thiogalactoside (IPTG) at OD of 0.4–0.6 at 600 nm. After overnight incubation at 25 °C and 80 rpm the cells were harvested by centrifugation (30 min, 7000 rpm, 4 °C). Sonification (3 times for 30 s, 1 min break, on ice) was applied to disrupt 5 g cells re-suspended in threefold volume of lysis-buffer (20 mM NaH_2_PO_4_, 300 mM NaCl, 5 mM imidazole, pH 8.0). After centrifugation (30 min, 15,000× *g*), the crude extract was heated in two steps (30 min 75 °C, 30 min 85 °C) as described by Wanarska *et al.* [28]. Following a second centrifugation step and filtration (0.8 μm membrane), the crude extract was loaded onto a HisTrap™ HP column (GE-Healthcare, Munich, Germany) (5 mL). Isolation of the N-terminal His-tagged glycosidase was accomplished by increasing the concentration of imidazole stepwise to 250 mM for elution (wash-buffer: 20 mM NaH_2_PO_4_, 300 mM NaCl, 20 mM imidazole pH 8.0; elution buffer: 20 mM NaH_2_PO_4_, 300 mM NaCl, 250 mM imidazole, pH 8.0). For further utilization of the enzyme, the elution buffer was exchanged by 25 mM citrate-Na_2_HPO_4_, pH 5.5.

### 3.3. Determination of Enzymatic Activities

Hydrolytic activity of enzyme solutions was monitored in 96-well microplate using the Synergy2 spectrophotometer (BioTek, BadFriedrichshall, Germany). The standard assay was performed at 85 °C for 5 min in a heating block containing 50 μL diluted protein solution (dilution appropriate to the calibration curve) and 450 μL aryl-substrates (30 mM *p*NP-β-d-Gal, 30 mM *p*NP-β-d-Glc, 15 mM *p*NP-β-d-Man, 30 mM *pNP*-β-d-Xyl, 30 mM *o*NP-β-d-Gal in 25 mM citrate/Na_2_HPO_4_-buffer, pH 5.5). Both solutions were combined after a 1 min pre-incubation at 85 °C. At specified intervals, the enzymatic reaction was stopped by addition of a 100 µL reaction mixture to 200 µL 0.2 M Na_2_CO_3_ at room temperature. Released nitrophenol was monitored at 405 nm. Calculation of enzyme units, defined as 1 µmol released nitrophenol per min for one unit, was done via calibration curves with *p*NP or *o*NP. Bradford assay (BSA as reference) was utilized to determine the protein concentration according to standard protein assay.

For comparison of experiments testing, hydrolytic performance under thermal heating (TH) in a conventional heating device and microwave irradiation (MWI) in a Discover^®^CoolMate™ system (CEM, Kamp-Lintfort, Germany), enzymatic cleavage of *p*NPGal was analyzed without the initial step of pre-incubation of both solutions. Reactions in the microwave reaction vessel were scaled to a volume of 1 mL (100 μL enzyme and 900 μL substrate solution). Control reactions without enzyme solution were run in parallel for each reaction condition.

Enzyme stability measurements at different microwave irradiation power (100 and 300 W) and at high temperature (85 °C), respectively, were performed with 1 mL diluted enzyme solution (~5 µg/mL) in a standard activity assay with *p*NPGal as described above. Samples were analyzed for residual hydrolytic activity applying standard conditions in the heating block with a set pre-incubation time. The volumetric activity determined under standard conditions in a heating block without a long-lasting heat treatment was set as a reference (100%).

### 3.4. Microwave System Equipment

Discover^®^CoolMate™ (CEM, Kamp-Lintfort, Germany) was employed for all performed microwave reactions. Galden HT-55 PTFE liquid (Solvay Solexis, Solvay Specialty Polymers Italy) was used as heat transfer fluid. This irradiation transparent solution was quickly cooled down by liquid nitrogen. For all experiments, the fixed power program (100, 200 or 300 W) was chosen. Online monitoring of temperature was realized with a fiber optic probe. The best initial temperatures for the cooling fluid were −60 °C for reactions at highest power (300 W) or −40 °C for fixed conditions at 100 and 200 W, respectively.

### 3.5. Synthesis of Glycoconjugates

Transgalactosylation reactions were performed with 10 or 50 mM acceptor substrate (GlcNAc-linker-*t*Boc), respectively, and 600 mM lactose, were dissolved in 25 mM citrate–Na_2_HPO_4_-buffer, pH 5.5. Reactions were started with 12 U/mL (~0.1 µg/mL) or 60 U/mL, respectively, purified recombinant β-galactosidase and incubated in a thermostat device at 12, 20, 30, or 85 °C, respectively, or in Discover^®^CoolMate™ (100 or 300 W) as indicated. Samples of the reaction solutions were taken at particular time intervals, stopped directly with three-fold volumetric excess of cooled ethanol, and stored on ice or at −20 °C before HPLC product analysis. Samples were diluted with water (final concentration of acceptor substrate < 5 mM) for analytical HPLC analysis.

### 3.6. Product Analysis and Characterization

Reversed-phase HPLC coupled to a UV/VIS detector was used for product analysis as previously described [33]. Following centrifugation for 10 min at 13,000 rpm samples were loaded onto a LiChrospher^®^ 100 RP 18 column. Selected purified samples were also analyzed by HPLC/ESI MS and NMR spectroscopy as previously described [36]. 

NMR analysis of the main product Gal(β1,4)GlcNAc-linker-*t*Boc was in accordance with previously published data [37]: **^1^**H NMR (400 MHz, D**_2_**O) δ = 4.46 (d, *J* = 7.8 Hz, 1H, H-1B), 3.83–3.97 (m, 4H, 2H-6A, H-2A, H-4A), 3.83–3.69 (m, 6H, 2H-6B, H-3A, H-4A, H-1, H-5B), 3.69–3.58 (m, 3H, H-2B, H-5A, H-3B), 3.53 (dd, *J* = 10.0, 7.7 Hz, 1H, H-2B), 3.24 (m, 2H), 2.00 (s, 3H, 2A-Ac), 1.41 (s, 9H, (CH**_3_**)3C)). The spectrum is depicted in Figure S16 in supporting information.

## 4. Conclusions

We here demonstrate the proof-of-concept for the use of microwave irradiation in biocatalytic transglycosylation reactions. MWI turned out to be favorable for transgalactosylation reactions utilizing the hyperthermophilic β-glycosidase from *Pyrococcus* at temperatures far below its temperature optimum. Nearly the same product yields can be obtained due to lack or delayed product hydrolysis under MWI. Furthermore, MWI promotes the synthesis of defined regioisomers facilitating subsequent product isolation. Our MWI strategy could be used as a novel experimental set-up for the synthesis of defined mixtures of galacto-oligosaccharides (GOS) from lactose. A detailed product analysis was not performed in this study. Work is in progress to exploit MWI in glycosylation reactions employing an active site engineered hyperthermophilic glycosidase (glycosynthase) at low temperatures suitable for the heat-labile glycosyl-fluoride donor substrates.

## Supplementary Materials

Supplementary materials can be found at http://www.mdpi.com/1422-0067/17/2/210/s1.
