# Supplementary material for: Microwave-Assisted Synthesis of Glycoconjugates by Transgalactosylation with Recombinant Thermostable β-Glycosidase from Pyrococcus
Source: Int J Mol Sci. 2016 Feb 4;17(2):210. doi: 10.3390/ijms17020210 (PMC4783942; doi:10.3390/ijms17020210)
Supplement: Supplementary file 1 [file ijms-17-00210-s001.pdf]

Supplementary Materials: Microwave-Assisted Synthesis of Glycoconjugates by Transgalactosylation with Recombinant Thermostable β-Glycosidase from *Pyrococcus*

Manja Henze, Dorothee Merker and Lothar Elling

1. Characteristics of the Recombinant β-Glycosidase from Pyrococcus

1.1. Gene Sequencing/Data Bank Screening

```
AF043283.1        1 mfpekflwgvaqsgfqfemgdklrrnidtntdwwhwvrdktniekglvsgdlpeeginny
E08095.1          1 ............................................................
AAC44387.1        1 ............................................................

AF043283.1       61 elyekdheiarklglnayrigiewsrifpwpttfidvdysynesynliedvkitkdtlee
E08095.1         61 ............................................................
AAC44387.1       61 ............................................................

AF043283.1      121 ldeiankrevayyrsvinslrskgfkvivnlnhftlpywlhdpieareraltnkrngwvn
E08095.1        121 ............................................................
AAC44387.1      121 ............................................................

AF043283.1      181 prtviefakyaayiaykfgdivdmwstfnepmvvvelgylapysgfppgvlnpeaaklai
E08095.1        181 ............................................................
AAC44387.1      181 ............................................................

AF043283.1      241 lhminahalayrqikkfdtekadkdskepaevgiiynnigvaypkdpndskdvkaaendn
E08095.1        241 ............................................................
AAC44387.1      241 ............................................................

AF043283.1      301 ffhsglffeaihkgklniefdgetfidapylkgndwigvnyytrevvtyqepmfpsipli
E08095.1        301 ............................................................
AAC44387.1      301 ............................................................

AF043283.1      361 tfkgvqgygyacrpgtlskddrpvsdigwelypegmydsiveahkygvpvyvtengiads
E08095.1        361 ............................................................
AAC44387.1      361 ............................................................

AF043283.1      421 kdilrpyyiashikmtekafedgyevkgyfhwaltdnfewalgfrmrfglyevnlitker
E08095.1        421 ...............i............................................
AAC44387.1      421 ...............i............................................

AF043283.1      481 ipreksvsifreivanngvtkkieeellrg
E08095.1        481 ..............................
AAC44387.1      481 ..............................
```

Figure S1. Alignment of protein sequences from different gene bank entries. The accession number AF043283.1 corresponds to β-galactosidase from Pyrococcus woesei (DSM 3773) [25]. The accession number AAC44387.1 corresponds to β-mannosidase from Pyrococcus furiosus (DSM 3638) [30]. The accession number E08095.1 corresponds to β-galactosidase from Pyrococcus furiosus [25]. The difference at position 436 is highlighted.

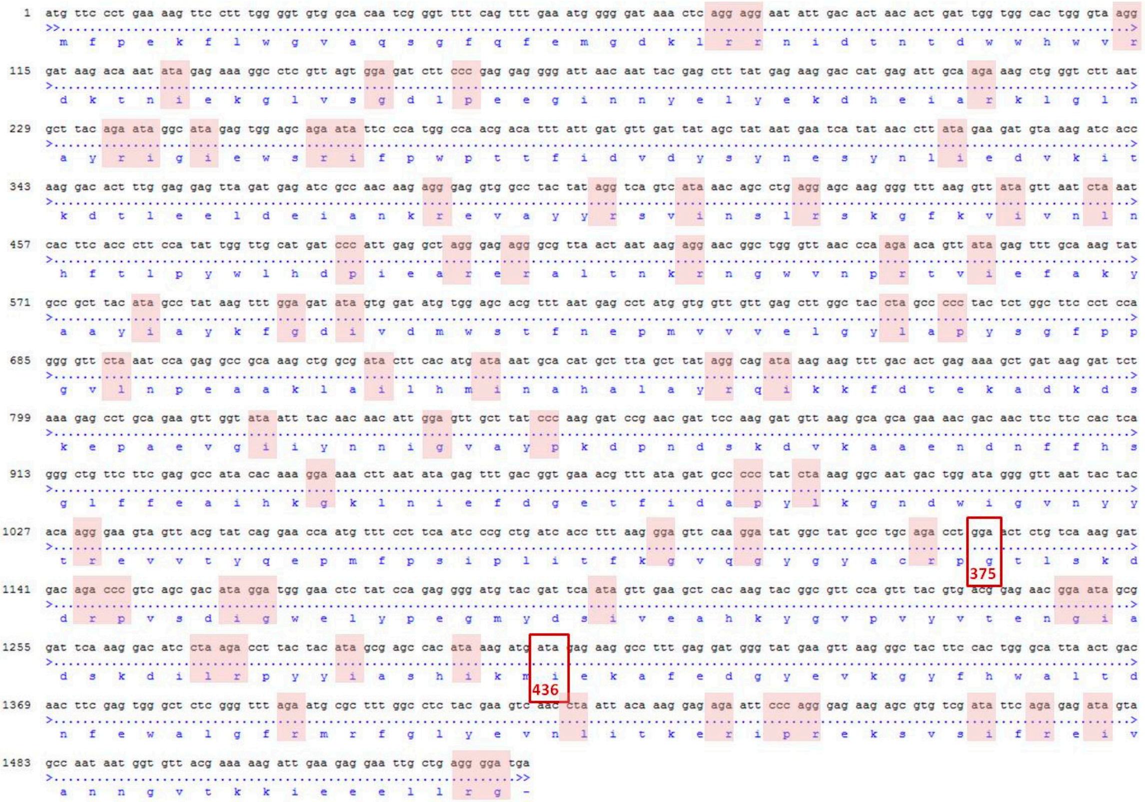

Figure S2. Sequenced gene of cloned pET-Duet™-1-His$_6$βGal. In comparison to the published β-galactosidase sequence from *Pyrococcus woesei* (GenBank: AF043283.1) two nucleotide sequences were different at amino acid positions 375 (glycine to glycine) and 436 (threonine to isoleucine) (red box). *E. coli* Rosetta 2 strain support the expression of rare codons used in *E. coli* (highlighted codons).

1.2. IMAC Purification and SDS-PAGE Analysis of Recombinant Protein

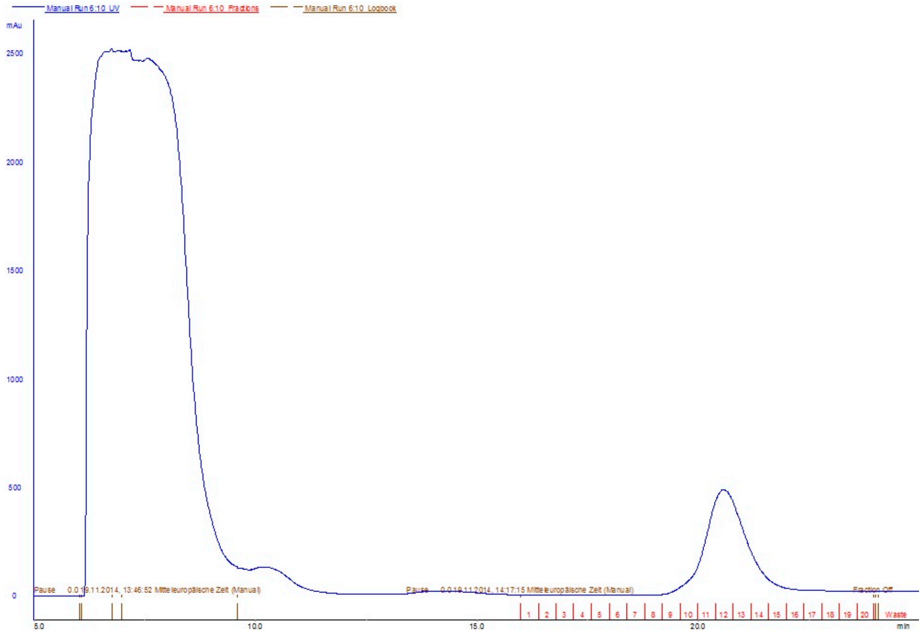

Figure S3. IMAC chromatogramm of crude extract containing soluble recombinant β-glycosidase after heat treatment. 5g Rosetta2™(DE3)pLysS cells were resuspended in 15 mL lysis buffer, sonicated and partially denatured (30 min 75 °C, 30 min 85 °C) before loading on 5 mL HisTrap HP column. UV-Signal was monitored at 280 nm.

Fractions of each purification step were checked on 10% SDS-PAGE. Gels were stained with Coomassie Brilliant Blue (Figure S4). SDS-gels showed the presence of overexpressed β-galactosidas from *Pyrococcus*.

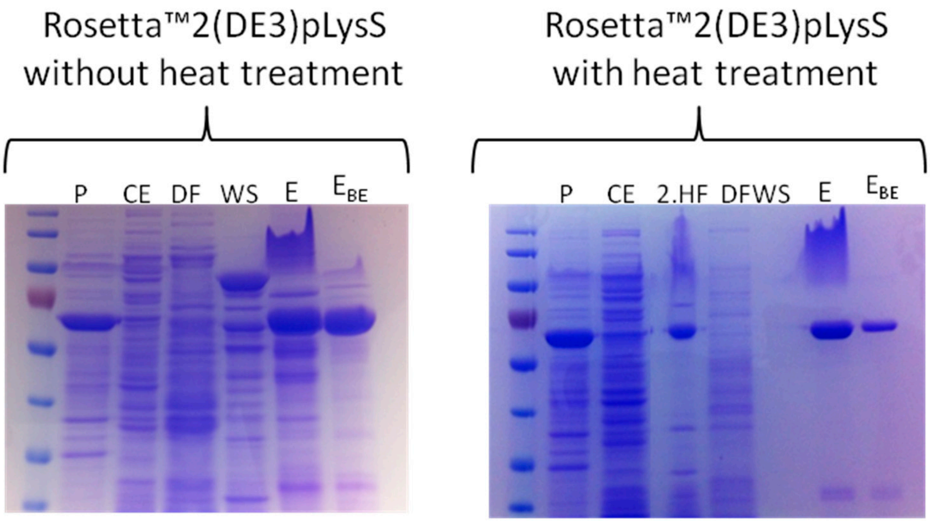

Figure S4. SDS-PAGEs of expression strain Rosetta2™(DE3)pLysS during different methods of protein isolation (61 kDa). PageRuler Prestained Protein Ladder (Fermentas) was used as reference standard. P: pellet, CE: crude extract, DF: Flow-through, WS: wash step, 2.HF: crude extract after second heat step, E: elution and EBE: elution after buffer exchange.

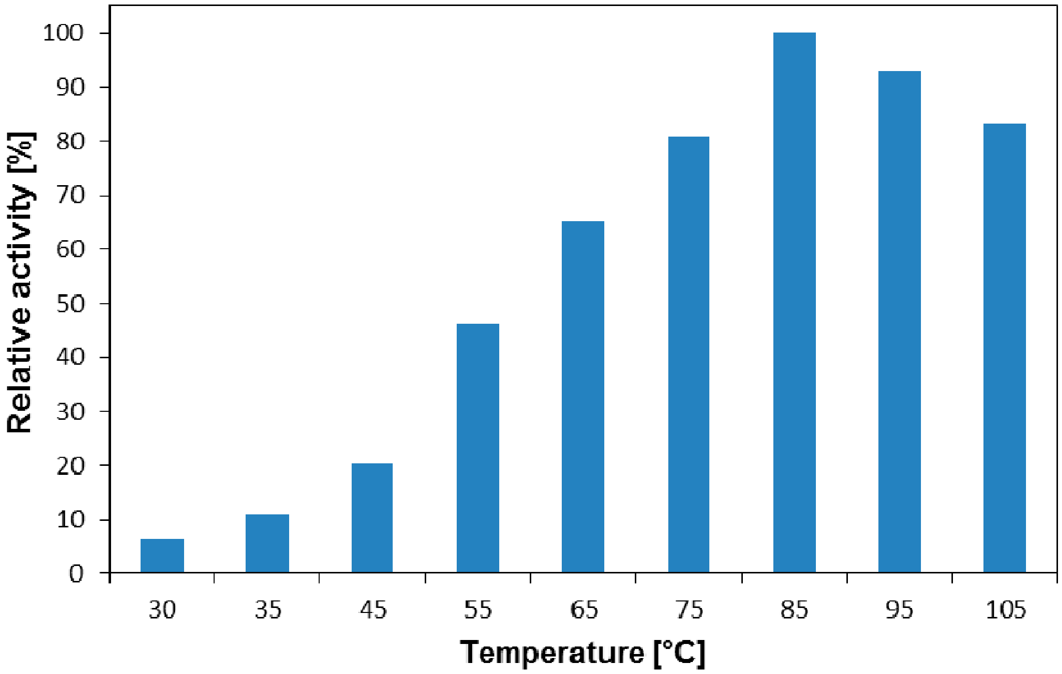

Figure S5. Effect of temperature on recombinant galactosidase activity, measured by hydrolysis of 30 mM pNPGal. 100% corresponds to 18 U/mL.

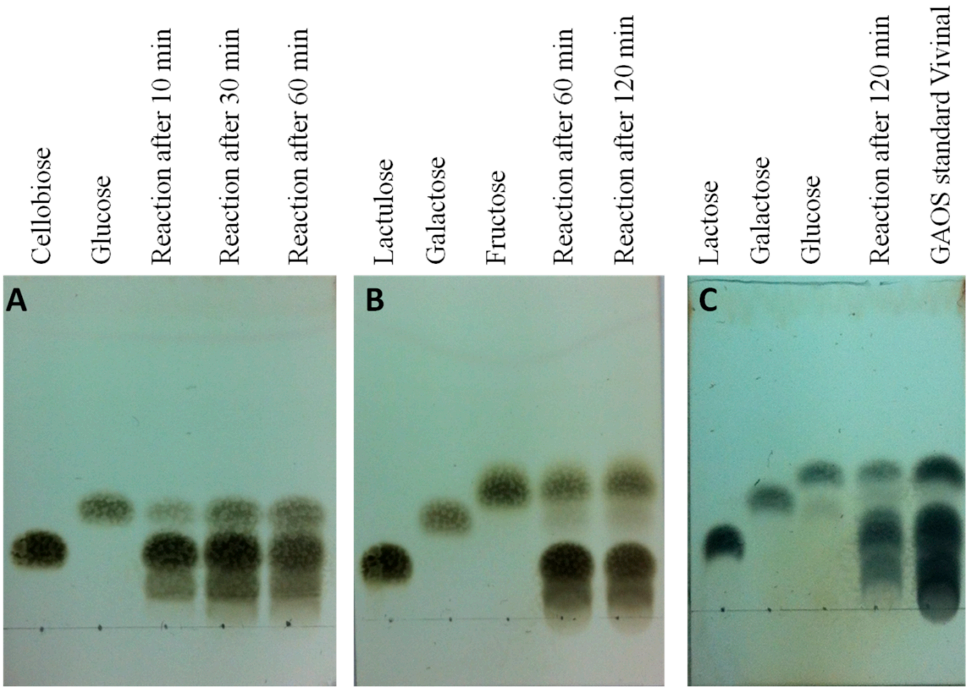

Figure S6. TLC analysis of hydrolysis and transglycosylation reactions with recombinant β-glycosidase (8 U/mL) from *Pyrococcus* and different disaccharides (600 mM) (**A**) cellobiose; (**B**) lactulose; (**C**) lactose. Reactions were stopped with three-fold access of cooled ethanol and stored on ice before TLC analysis. 6 µL of 20 mM standard sugars and 6 µL synthesis probes (dilution 1:6) were loaded on Alugram®Xtra SIL G plates (Macherey-Nagel, Düren, Germany). Samples were analysed by treatment with propane-2-ol/2 water/ammonia (7:2:1) and detected with 10% sulphuric acid in ethanol as previously reported by Slamova *et al.* [38]. GAOS Vivinal was used as standard of galacto-oligosaccharides.

1.3. Specificity of Recombinant β-Glycosidase for the Hydrolysis of the Regioisomers Galβ(1,3/4/6)GlcNAc-Linker-tBoc

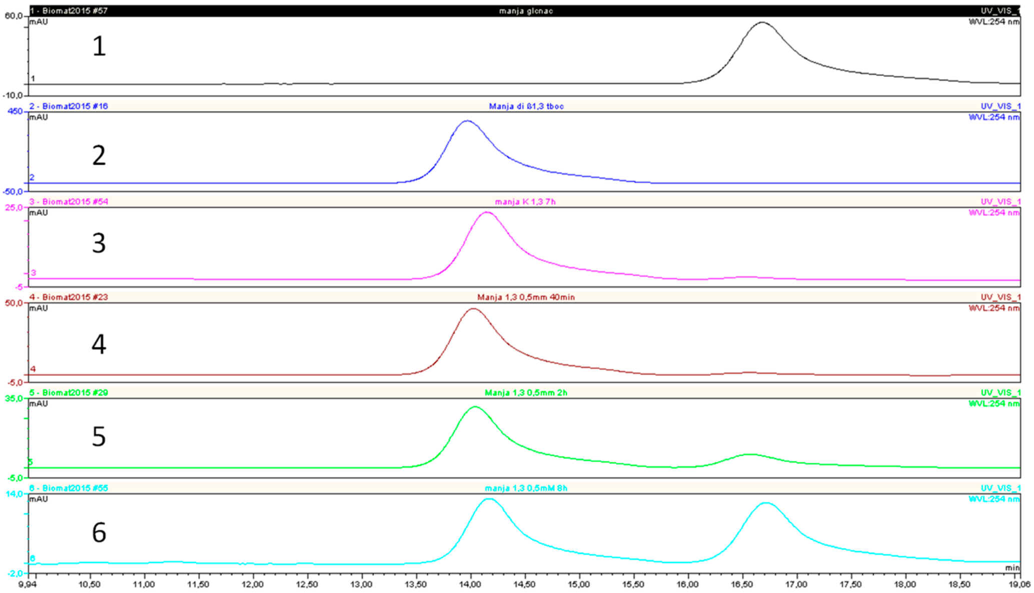

Figure S7. Enzymatic hydrolysis of Galβ(1,3)GlcNAc-linker-*t*Boc. Substrate and product were monitored by RP-HPLC at 254 nm: GlcNAc-linker-*t*Boc (1), Galβ(1,3)GlcNAc-linker-*t*Boc (2), reaction without enzyme after 7 h (3), reaction after 40 min (4), reaction after 2 h (5) and reaction after 8 h (6). Conditions: 0.5 mM disaccharide and 20 U/mL enzyme solution were incubated at 85 °C.

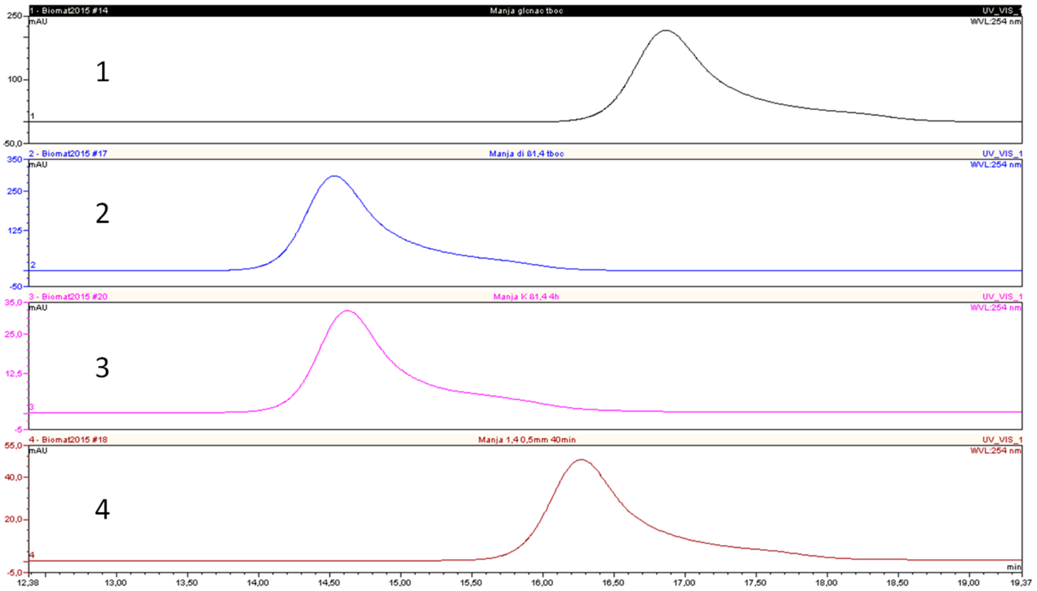

Figure S8. Enzymatic hydrolysis of Galβ(1,4)GlcNAc-linker-*t*Boc. Substrate and product were monitored by RP-HPLC at 254 nm: GlcNAc-linker-*t*Boc (1), verified Galβ(1,4)GlcNAc-linker-*t*Boc (2), reaction without enzyme after 4 h (3) and reaction after 40 min (4). Conditions: 0.5 mM disaccharide and 20 U/mL enzyme solution were incubated at 85 °C.

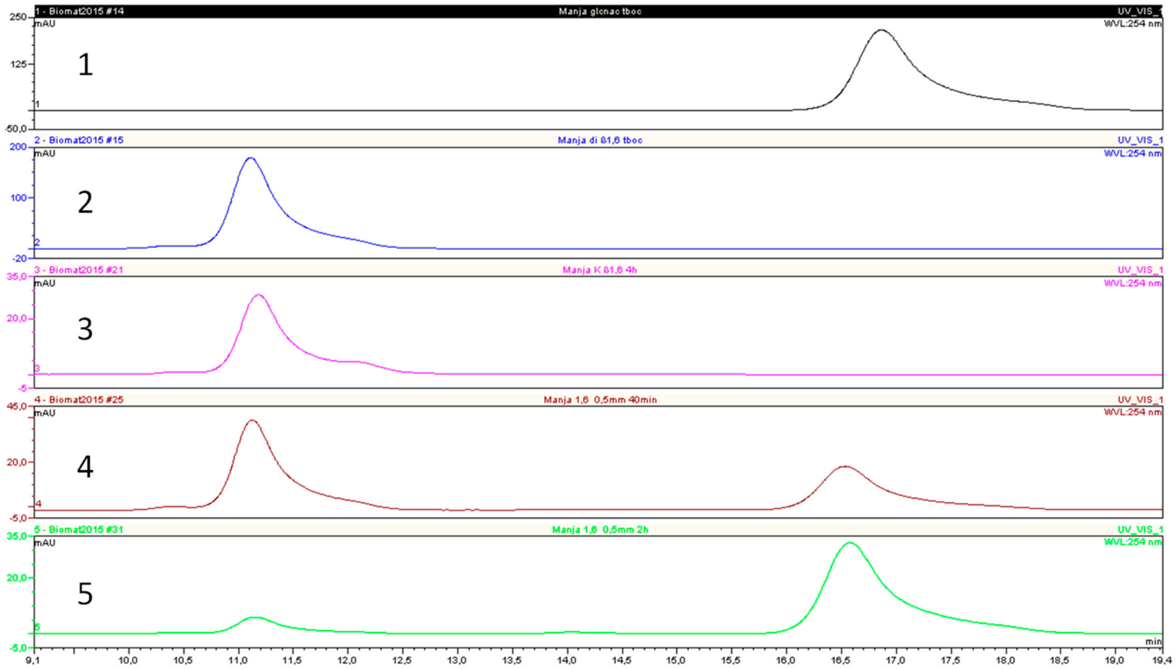

Figure S9. Enzymatic hydrolysis of Galβ(1,6)GlcNAc-linker-*t*Boc. Substrate and product were monitored by RP-HPLC at 254 nm: GlcNAc-linker-*t*Boc (1), verified Galβ(1,6)GlcNAc-linker-*t*Boc (2), reaction without enzyme after 4 h (3); reaction after 40 min (4) and reaction after 2 h (5). Conditions: 0.5 mM disaccharide and 20 U/mL enzyme solution were incubated at 85 °C.

1.4. Hydrolytic Activity of Recombinant β-Glycosidase from Pyrococcus under TH and MWI

Table S1. Comparison of different reaction conditions in Microwave™ Synthesis System with stirring (high) of the reaction solutions. Hydrolytic β-galactosidase activity under fixed microwave energy (MWI) in comparison to conventional thermal heating (TH) was investigated. Release of *p*NP from 30 mM *p*NPGal in 25 mM citrate-phosphate buffer pH 5.5 was continuously monitored by spectrophotometric assay. ΔT depicts the variation in temperature measured within the reaction vessel by the fiber optic sensor. 100% corresponds to 3.4 U/mL.

Reaction Condition	Relative Activity (%)	Max. Temperature (°C)
MWI 100 W	36	12 °C (ΔT = 4 °C)
MWI 100 W + stirring	32	22 °C (ΔT = 6 °C)
MWI 200 W	87.4	14 °C (ΔT = 4 °C)
MWI 200 W + stirring	74.3	23 °C (ΔT = 3 °C)
MWI 300 W	100	32 °C (ΔT = 12 °C)
MWI 300 W + stirring	86	27 °C (ΔT = 9 °C)

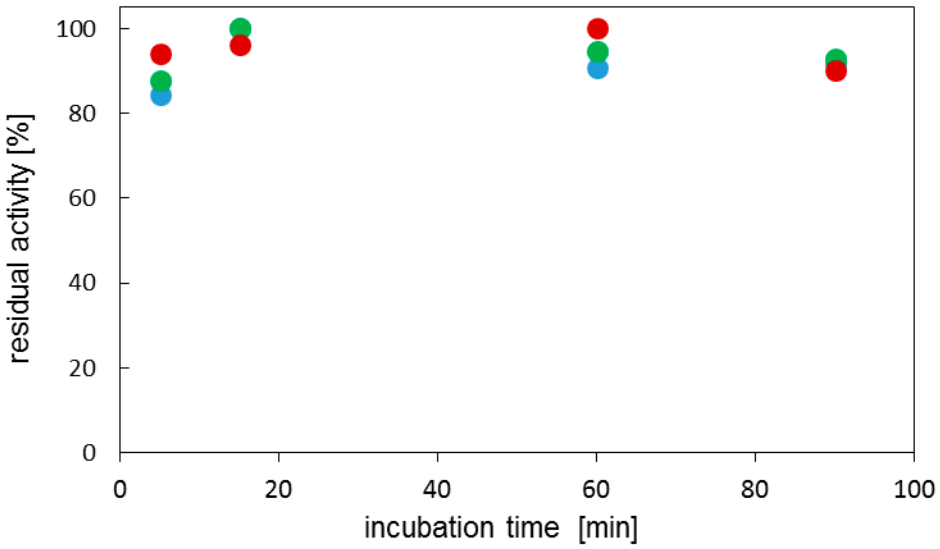

Figure S10. Effect of sucrose (1 M: green; 0.5 M: red) and the ionic liquid (IL) [BMIM][PF6] (blue) on the enzyme stability under thermal heating at 85 °C. Solutions with the ionic liquid included 450 µL IL, 1020 µL 25 mM citrate-phosphate buffer pH 5.5 and 30 µL diluted enzyme solution. 10 µL diluted enzyme solution were incubated with 490 µL 1 M or 0.5 M sucrose. The residual activity was measured with *p*NP-Gal under standard assay conditions.

2. Transglycosylation Reactions with Recombinant β-Glycosidase from *Pyrococcus* under Thermal Heating (85 °C)

2.1. Kinetic Analysis with Acceptor β-D-GlcNAc-Linker-tBoc

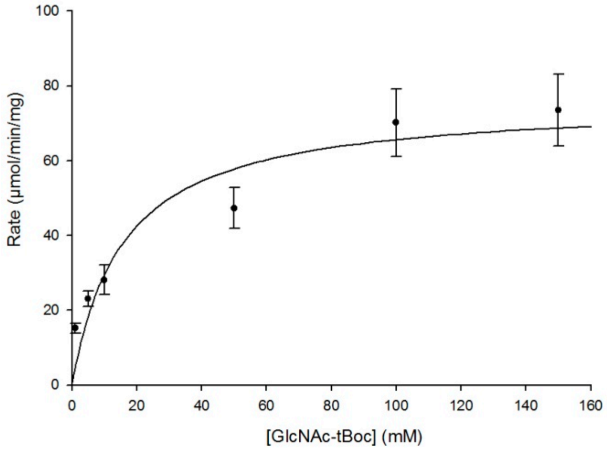

Figure S11. Michaelis-Menten graph for the transgalactosylation reaction of recombinant β-glycosidase from *Pyrococcus* under conventional thermal heating (85 °C). Enzyme activity was measured by monitoring (RP-HPLC) the disaccharide product Galβ(1,4)GlcNAc-linker-*t*Boc by variation of acceptor substrate concentrations of β-D-GlcNAc-linker-*t*Boc at constant lactose (600 mM) concentrations.

To determine the kinetic parameter K_m and v_{max} for the transglycosylation reaction with GlcNAc-linker-*t*Boc as acceptor substrate, the concentration was varied in the following steps: 1, 5, 10, 50, 100 and 150 mM. Lactose was used as donor substrate at a concentration of 600 mM. The formation of products was followed by reversed HPLC at 254 nm as described in material and methods (manuscript). The initial rate of substrate conversion was calculated from the percentage of formed disaccharide Galβ(1,4)GlcNAc-linker-*t*Boc. Michaelis-Menten kinetics were calculated by non-linear regression analysis using Sigma Plot 10 software (SPSS Science Software GmbH, Erkrath, Germany).

2.2. Isolation of Product Fractions and Characterization by HPLC/ESI-MS

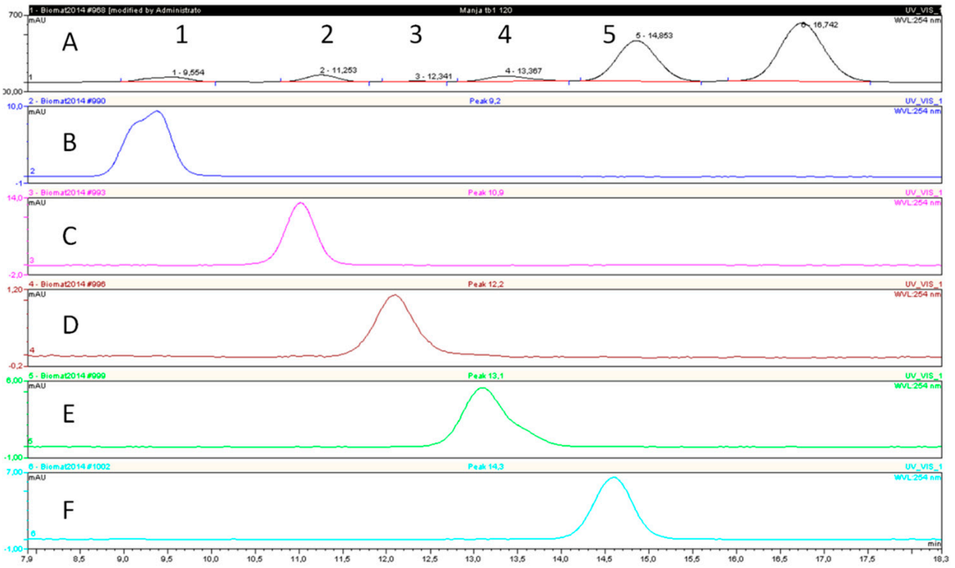

Figure S12. The product peaks 1–5 (**A**) from a preparative HPLC separation were isolated (**B** product 1; **C** product 2; **D** product 3; **E** product 4; and **F** product 5) and analyzed by ESI-MS measurements (Figure S13).

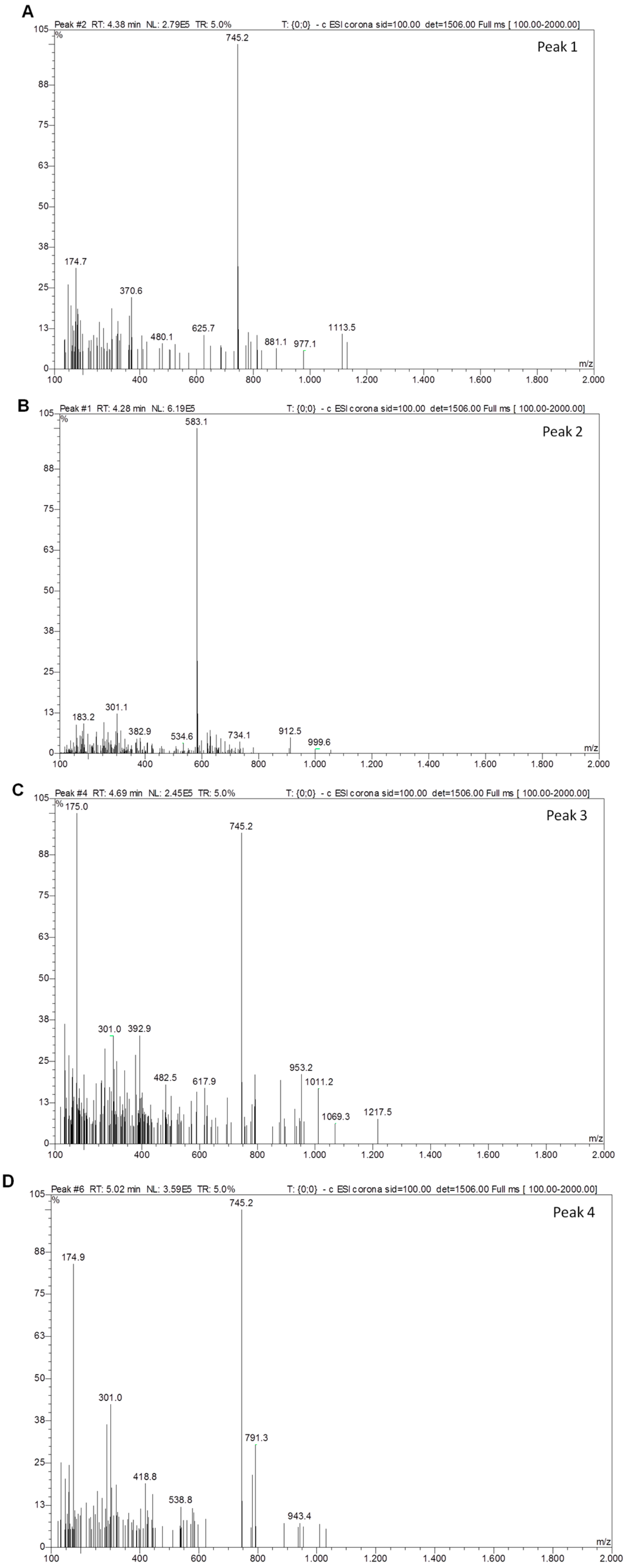

Figure S13. *Cont.*

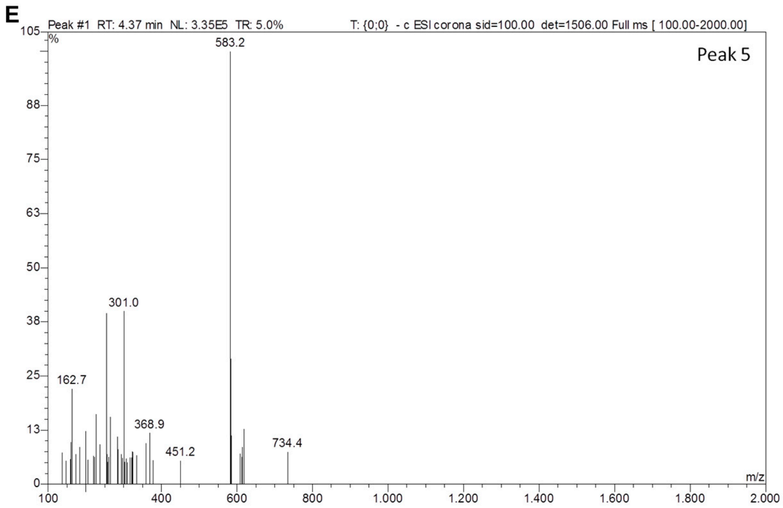

Figure S13. ESI-MS data of isolated products (Figure S11, product peaks 1–5) synthesized under thermal heating condition at 85 °C. Mass spectra of (**A**) **Peak 1**, found 745.2 for [M − H]⁻, calculated 746.7 for [M] for trisaccharide (Gal-Gal-GlcNAc-linker-*t*Boc); (**B**) **Peak 2**, found 583.1 for [M − H]⁻, calculated 584.6 for [M] for disaccharide (Gal-GlcNAc-linker-*t*Boc); (**C**) **Peak 3**, found 745.2 for [M − H]⁻, calculated 746.7 for [M] for trisaccharide (Gal-Gal-GlcNAc-linker-*t*Boc); (**D**) **Peak 4**, found 745.2 for [M − H]⁻, calculated 746.7 for [M] for trisaccharide (Gal-Gal-GlcNAc-linker-*t*Boc) and (**E**) **Peak 5** found 583.2 for [M − H]⁻, calculated 584.6 for [M] for disaccharide (Gal-GlcNAc-linker-*t*Boc).

2.3. Identification of the Main Product in Transgalactosylation Reactions of Recombinant β-Glycosidase from P. Woesei under Conventional Thermal Heating (85 °C)

Comparison with disaccharide regioisomers

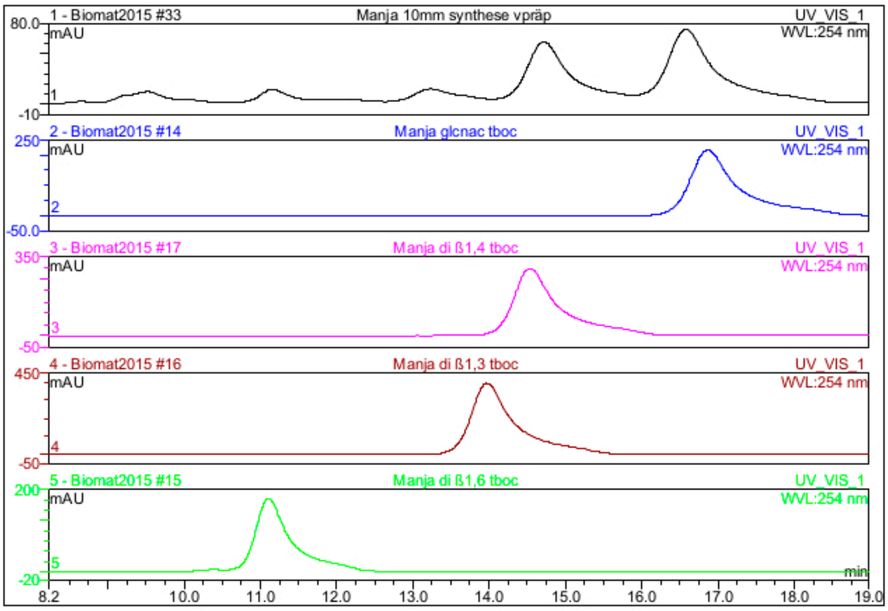

Figure S14. The product mixture (**1**) obtained by a transgalactosylation reaction (10 mM β-D-GlcNAc-linker-*t*Boc, 600 mM lactose with 12 U/mL) of recombinant β-glycosidase from *Pyrococcus* was compared with synthesized regioisomeric disaccharide standards. Separation was achieved on a LiChrospher® 100 RP 18 column with UV detection at 254 nm. (**1**) product mixture catalyzed by Pyrococcus β-D-galactosidase under thermal heating (85 °C) (**2**) GlcNAc-linker-tBoc, (**3**) Galβ(1,4)GlcNAc-linker-tBoc, (**4**) Galβ(1,3)GlcNAc-linker-*t*Boc and (**5**) Galβ(1,6)GlcNAc-linker-tBoc. The main product peak elutes at the same retention time as the standard **3**. A mixture of β(1,3/4) regioisomers is not formed.

2.4. Enzymatic Digestion

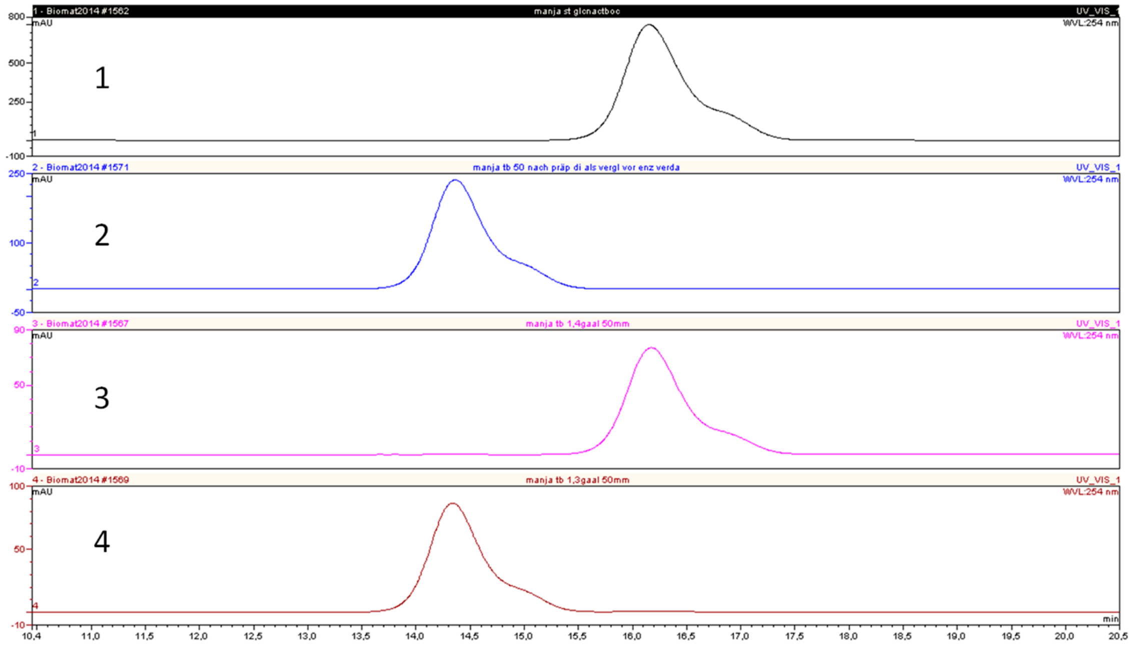

Figure S15. Enzymatic digestion of the main disaccharide product peak with commercial galactosidases β1,4 galactosidase S (New England Biolabs) and β1,3 galactosidase (New England Biolabs). Reactions were analysed by RP-HPLC as described above: GlcNAc-linker-*t*Boc (**1**), isolated disaccharide (**2**), digestion of main disaccharide product with β1,4 galactosidase S (**3**) and reaction with β1,3 galactosidase (**4**). The isolated main disaccharide product is cleaved by β1,4 galactosidase S. We conclude that Galβ(1,4)GlcNAc-linker-*t*Boc is the main product of the transgalactosylation reaction.

2.5. NMR-Analysis of the Main Product in Transgalactosylation Reactions of Recombinant β-Glycosidase From Pyrococcus under MWI and Conventional Thermal Heating (85 °C)

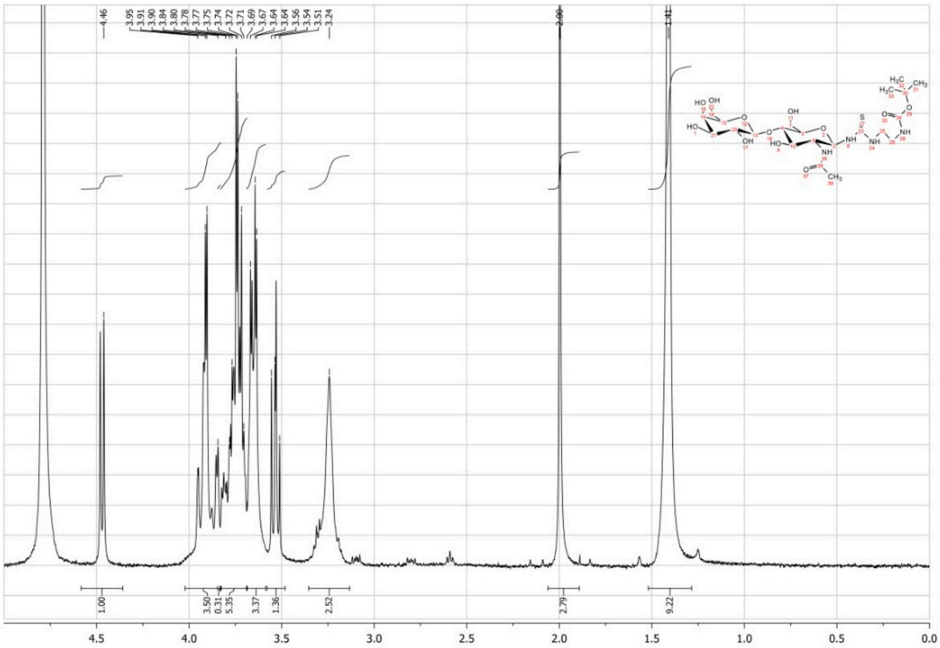

Figure S16. NMR spectrum of the major product obtained in microwave-assisted reaction with 50 mM acceptor substrate. Sample were purified by semi-preparative HPLC. NMR spectroscopy confirmed the main product as Galβ(1,4)GlcNAc-linker-*t*Boc. Identical result was obtained for the transgalactosylation reaction under thermal heating (85 °C).

2.6. Transgalactosylation Reactions of Recombinant β-Glycosidase from Pyrococcus under Conventional Thermal Heating (85 °C)

2.6.1. Transgalactosylation Reactions with 10 mm Acceptor Substrate β-D-Glcnac-Linker-*t*boc

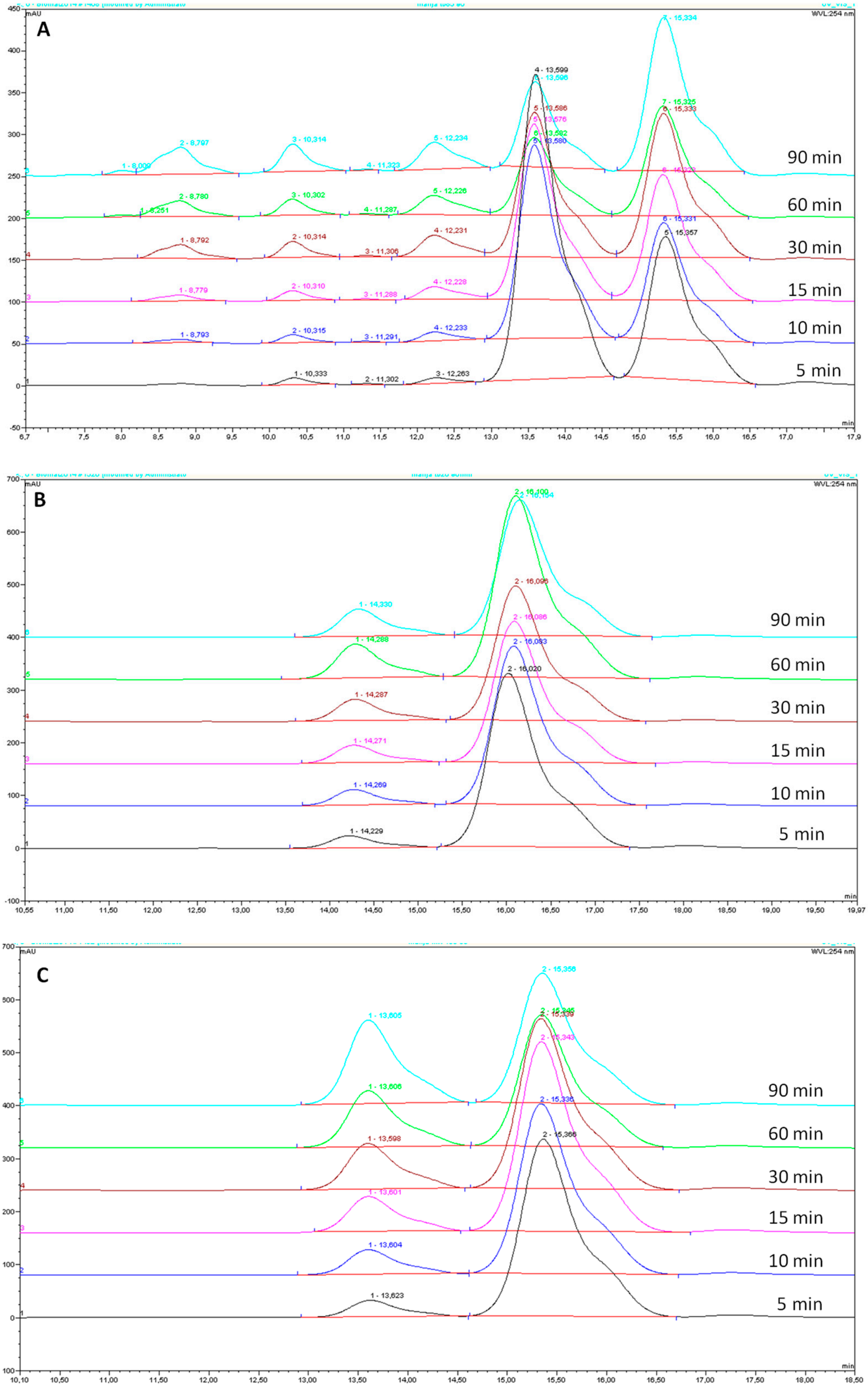

Figure S17. *Cont.*

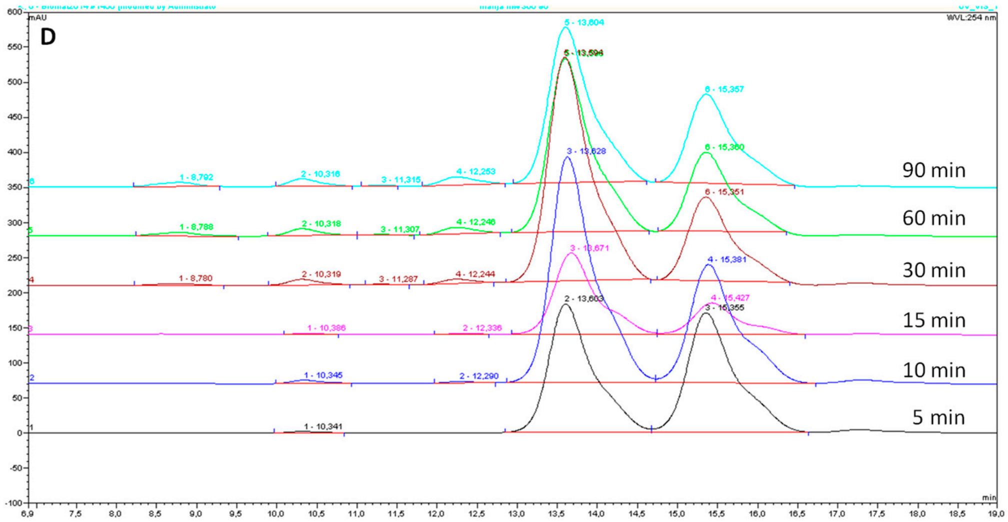

Figure S17. Transgalactosylation reactions with 10 mM GlcNAc-linker-*t*Boc as acceptor substrate (600 mM lactose) under various conditions with 12 U/mL enzyme solution (determined by hydrolysis of *p*NP-Gal). Syntheses were performed under thermal heating (TH) at 85 °C (**A**) and TH at 20 °C (**B**); as well as under MWI at 100 W (**C**) or 300 W (**D**).

Table S2. Relative amounts of single peaks from transgalactosylation reactions (10 mM GlcNAc-linker-*t*Boc and 600 mM lactose in 25 mM citrate-phosphate buffer pH 5.5) with 12 U β-glycosidase from *Pyrococcus* under microwave irradiation (MWI) and thermal heating (TH) at certain points. Due to the results of MS-analysis peaks can be assigned to different saccharides: **1** Gal-Gal-GlcNAc-linker-*t*Boc, **2** Gal-GlcNAc-linker-*t*Boc, **3** Gal-Gal-GlcNAc-linker-*t*Boc, **4** Gal-Gal-GlcNAc-linker-*t*Boc, **5** Galβ(1,4)GlcNAc-linker-*t*Boc and **6** GlcNAc-linker-*t*Boc (see Figure 2 and Table 3, manuscript).

	**Peak (%)**	**1**	**2**	**3**	**4**	**5**	**6**
**Condition**							
**5 min**	**TH 85 °C**	-	1.0	0.1	0.8	66.7	31.3
	**MWI 300 W**	-	0.3	-	-	49.0	50.6
**15 min**	**TH 85 °C**	1.8	2.1	0.3	3.5	52.0	40.4
	**MWI 300 W**	-	0.7	-	0.3	69.7	29.3
**60 min**	**TH 85 °C**	6.7	4.8	0.3	7.5	31.0	49.1
	**MWI 300 W**	1.0	1.9	0.2	1.7	64.4	30.8

2.7. Product Stability under MWI at 300 W

A transgalactosylation reaction (10 mM GlcNAc-linker-*t*Boc incubation under TH 85 °C, reaction time 90 min) was stopped by adding cooled ethanol. The product mixture was transferred to the microwave system and incubated at 300 W. Then samples of the mixture were analyzed by RP-HPLC at different time intervals. Figure S18 and Table S3 demonstrate the product stability under the maximum power input of 300 W.

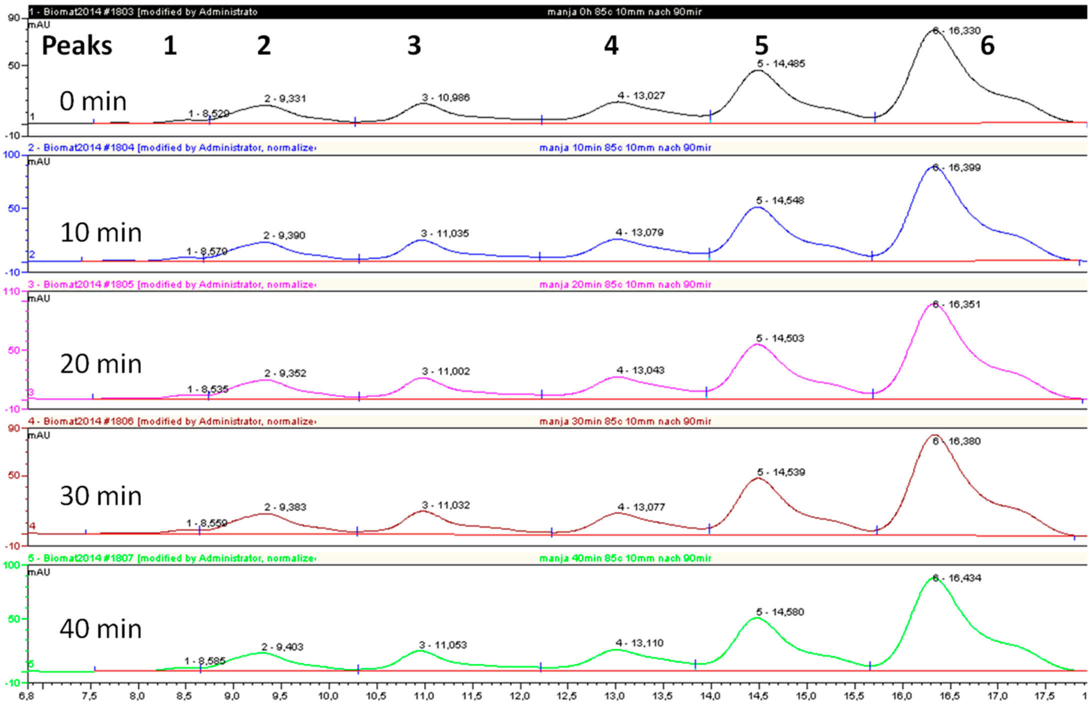

Figure S18. Stability of produced glycan structures under 300 W microwave power (see Table S3).

Table S3. Peak areas for a single peak at various points in time (see chromatogram Figure 2, Figure S18). Peaks 1–5 represent the products of transgalactosylation. Peak 6 depicts the reactant.

	Peak					
	1	2	3	4	5	6
**Incubation Time (min) at 300 W**	**Peak Area (mAU × min)**					
0	1.14	8.35	9.00	11.76	24.33	45.42
10	1.16	8.84	9.28	11.68	24.10	44.95
20	1.14	8.65	9.25	11.52	24.34	45.10
30	1.14	9.51	10.15	10.12	23.77	45.30
40	0.88	8.59	9.13	11.05	24.86	45.50

Table S4. Measurements with standard deviations (STD) for transgalactosylation reaction with hyperthermophilic β-glycosidase from *Pyrococcus* under MWI and TH starting from 10 mM GlcNAc-linker-*t*Boc (Figure 3).

**Reaction Time (min)**	**TH 85 °C**				**TH 20 °C**			
	**GlcNAc-Linker-*t*Boc**		**Gal(β1,4)GlcNAc-Linker-*t*Boc**		**GlcNAc-Linker-*t*Boc**		**Gal(β1,4)GlcNAc-Linker-*t*Boc**	
	**rel. Peak Area (%)**	**STD**	**rel. Peak Area (%)**	**STD**	**rel. Peak Area (%)**	**STD**	**rel. Peak Area (%)**	**STD**
0	100.0	0.00	0.0	0.00	100.0	0.00	0.0	0.00
5	31.3	1.83	66.7	1.71	94.9	0.30	5.1	0.30
10	37.4	0.37	57.4	0.43	92.6	0.04	7.4	0.04
15	40.4	0.19	52.0	0.77	90.5	0.19	9.5	0.19
30	45.3	0.33	41.5	1.19	88.1	0.01	11.9	0.01
60	49.1	0.10	31.0	1.28	86.2	0.12	13.8	0.12
90	51.7	0.64	24.4	1.01	85.4	0.04	14.6	0.04

Table S4. *Cont.*

Reaction Time (min)	MWI 300 W				MWI 100 W			
	GlcNAc-Linker-*t*Boc		Gal(β1,4)GlcNAc-Linker-*t*Boc		GlcNAc-Linker-*t*Boc		Gal(β1,4)GlcNAc-Linker-*t*Boc	
	rel. Peak Area (%)	STD	rel. Peak Area (%)	STD	rel. Peak Area (%)	STD	rel. Peak Area (%)	STD
0	100.0	0.00	0.0	0.00	100.0	0.00	0.0	0.00
5	50.1	0.78	49.0	0.87	93.2	0.66	6.8	0.66
10	35.9	1.12	64.1	1.20	89.8	1.17	10.2	1.17
15	28.9	0.57	69.7	0.72	87.9	1.93	12.1	1.93
30	27.2	0.41	69.9	0.15	82.2	1.83	17.8	1.83
60	30.5	0.46	64.4	0.15	74.4	2.99	25.6	2.99
90	36.1	0.00	58.4	0.00	68.3	6.98	31.7	6.98

2.8. Synthesis with 50 mM Acceptor Substrate β-D-GlcNAC-Linker-tBoc

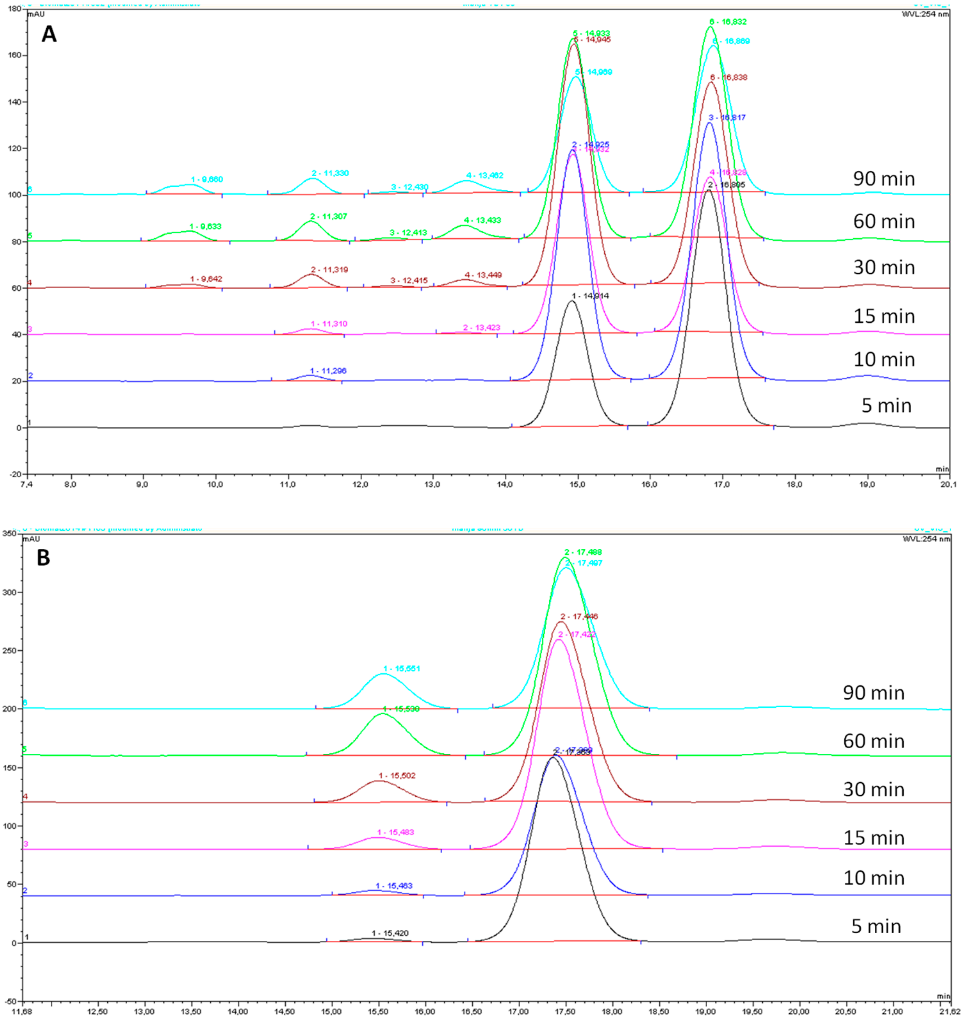

Figure S19. *Cont.*

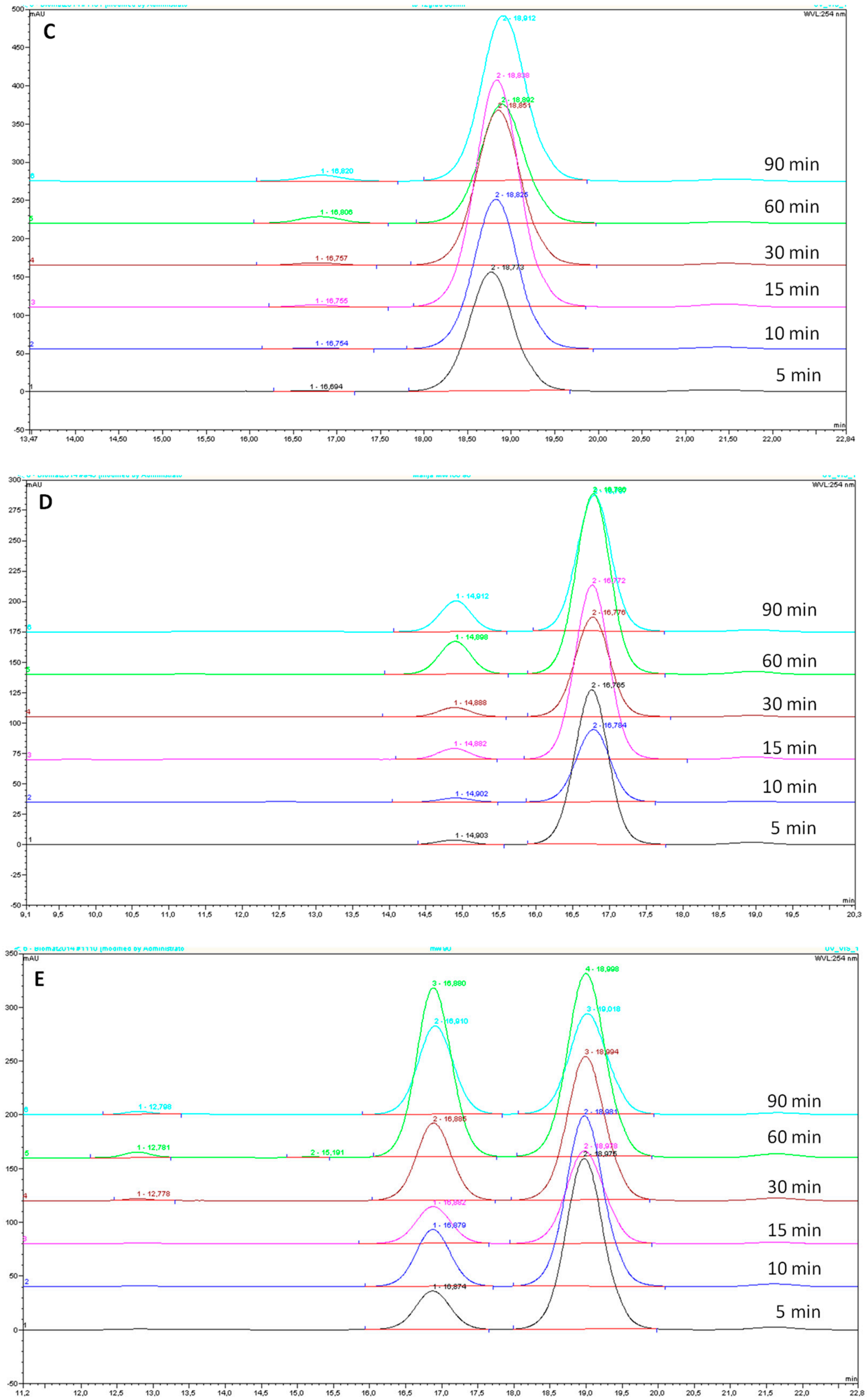

Figure S19. Transgalactosylation reactions with 50 mM GlcNAc-linker-*t*Boc as acceptor substrate under various conditions with 12 U/mL enzyme (determined by hydrolysis of *p*NP-Gal). Syntheses were performed under thermal heating at 85 °C (**A**) at 30 °C (**B**); and at 12 °C (**C**) as well as under MWI at 100 W (**D**) or microwave energy at 300 W (**E**).

Table S5: Relative amounts of single peaks from transgalactosylation reactions (50 mM GlcNAc-linker-*t*Boc and 600 mM lactose in 25 mM citrate-phosphate buffer pH 5.5) with 12 U β-glycosidase from *Pyrococcus* under microwave irradiation (MWI) and thermal heating (TH) at certain points. Due to the results of MS-analysis peaks can be assigned to different saccharides: **1** Gal-Gal-GlcNAc-linker-*t*Boc, **2** Gal-GlcNAc-linker-*t*Boc, **3** Gal-Gal-GlcNAc-linker-*t*Boc, **4** Gal-Gal-GlcNAc-linker-*t*Boc, **5** Galβ(1,4)GlcNAc-linker-*t*Boc and **6** GlcNAc-linker-*t*Boc (see Figure 2 and Table 3, manuscript).

	**Peak (%)**	**1**	**2**	**3**	**4**	**5**	**6**
**Condition**							
**15 min**	**TH 85 °C**	-	1.3	-	1.0	50.0	47.3
	**MWI 300 W**	-	0.1	-	-	22.3	77.6
**60 min**	**TH 85 °C**	3.2	3.5	0.2	3.8	36.8	52.5
	**MWI 300 W**	-	0.7	-	-	39.3	59.9

Table S6. Measurements with standard deviations (STD) for transgalactosylation reaction with hyperthermophilic β-glycosidase from *Pyrococcus* under MWI and TH starting from 50 mM GlcNAc-linker-*t*Boc (Figure 4).

**Reaction Time (min)**	**MW 100 W**				**MW 300 W**			
	**GlcNAc-Linker-*t*Boc**		**Gal(β1,4)GlcNAc-Linker-*t*Boc**		**GlcNAc-Linker-*t*Boc**		**Gal(β1,4)GlcNAc-Linker-*t*Boc**	
	**rel. Peak Area (%)**	**STD**	**rel. Peak Area (%)**	**STD**	**rel. Peak Area (%)**	**STD**	**rel. Peak Area (%)**	**STD**
0	100.0	0.00	0.0	0.00	100.0	0.00	0.0	0.00
5	96.0	1.55	4.0	1.55	85.7	4.01	14.3	4.01
10	94.0	1.26	6.0	1.26	81.8	3.93	18.2	3.93
15	92.9	2.14	7.1	2.14	77.6	3.48	22.3	3.50
30	87.8	4.81	12.2	4.82	70.8	4.40	28.9	4.26
60	82.4	4.34	17.6	4.34	59.9	9.87	39.3	9.62
90	81.4	7.16	18.6	7.16	61.1	9.37	38.4	8.62

**Reaction Time (min)**	**TH 85 °C**				**TH 30 °C**			
	**GlcNAc-Linker-*t*Boc**		**Gal(β1,4)GlcNAc-Linker-*t*Boc**		**GlcNAc-Linker-*t*Boc**		**Gal(β1,4)GlcNAc-Linker-*t*Boc**	
	**rel. Peak Area (%)**	**STD**	**rel. Peak Area (%)**	**STD**	**rel. Peak Area (%)**	**STD**	**rel. Peak Area (%)**	**STD**
0	100.0	0.00	0.0	0.00	100.0	0.00	0.0	0.00
5	59.5	7.93	40.4	7.81	98.2	0.36	1.8	0.36
10	49.9	4.51	48.7	3.83	96.8	0.65	3.2	0.65
15	47.3	0.27	50.0	1.23	95.1	0.47	4.9	0.47
30	48.6	3.93	45.3	5.91	89.9	0.78	10.1	0.78
60	52.5	4.07	36.8	6.60	84.9	1.07	15.1	1.07
90	54.2	3.81	31.4	7.22	80.5	1.06	19.5	1.06

**Reaction Time (min)**	**TH 12 °C**			
	**GlcNAc-Linker-*t*Boc**		**Gal(β1,4)GlcNAc-Linker-*t*Boc**	
	**rel. Peak Area (%)**	**STD**	**rel. Peak Area (%)**	**STD**
0	100.0	0.00	0.0	0.00
5	99.5	0.16	0.5	0.16
10	99.4	0.10	0.6	0.10
15	99.0	0.27	1.0	0.27
30	98.7	0.17	1.3	0.17
60	96.5	1.90	3.5	1.90
90	96.9	0.07	3.1	0.07

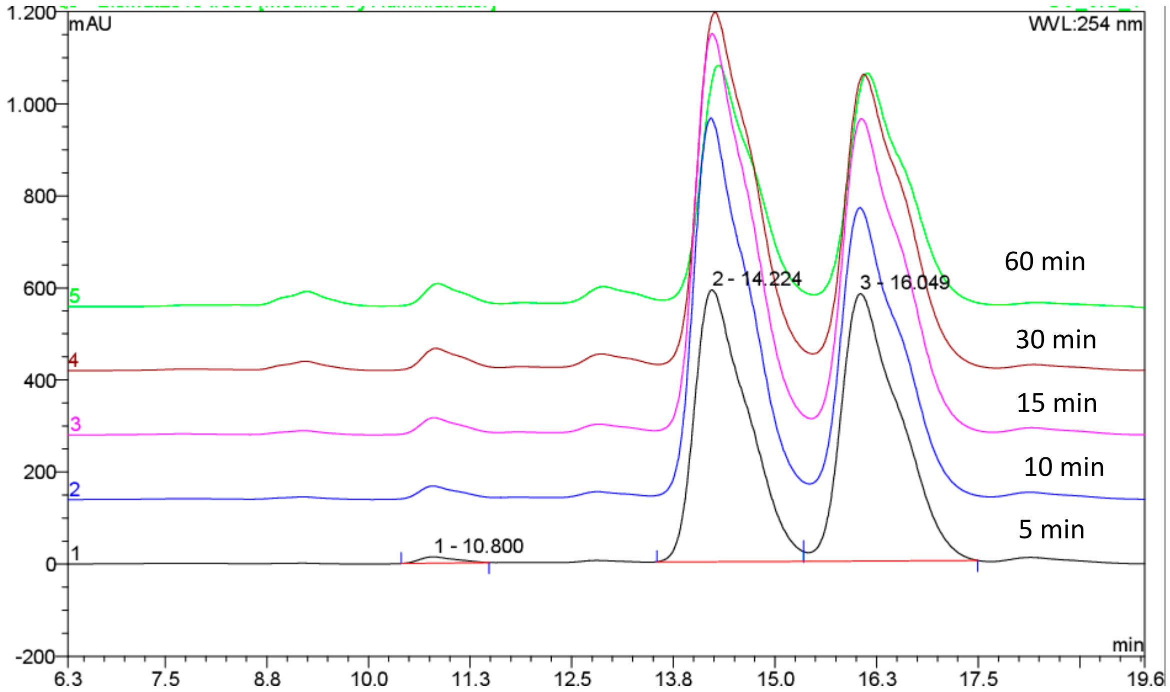

Figure S20. Transgalactosylation reactions with 50 mM GlcNAc-linker-*t*Boc as acceptor substrate under MWI at 300 W with 60 U/mL enzyme (determined by hydrolysis of *p*NP-Gal).

Table S7. Relative amounts of single peaks from transgalactosylation reactions (50 mM GlcNAc-linker-*t*Boc and 600 mM lactose in 25 mM citrate-phosphate buffer pH 5.5) with β-glycosidase from *Pyrococcus* under microwave irradiation (MWI, 60 U/mL) and thermal heating (TH, 12 U/mL) at certain points. Due to the results of MS-analysis peaks can be assigned to different saccharides: **1** Gal-Gal-GlcNAc-linker-*t*Boc, **2** Gal-GlcNAc-linker-*t*Boc, **3** Gal-Gal-GlcNAc-linker-*t*Boc, **4** Gal-Gal-GlcNAc-linker-*t*Boc, **5** Galβ(1,4)GlcNAc-linker-*t*Boc and **6** GlcNAc-linker-*t*Boc (see Figure 2 and Table 3, manuscript).

	**Peak (%)**	**1**	**2**	**3**	**4**	**5**	**6**
**Condition**							
**15 min**	**TH 85 °C**	-	1.3	-	1.0	50.0	47.3
	**MWI 300 W**	0.3	1.4	-	0.6	52.5	45.1
**60 min**	**TH 85 °C**	3.2	3.5	0.2	3.8	36.8	52.5
	**MWI 300 W**	2.0	2.9	-	2.1	44.7	48.2

Table S8. Measurements with standard deviations (STD) for transgalactosylation reaction with hyperthermophilic β-glycosidase from *Pyrococcus* under thermal heating at 85 °C with 12 U/mL in comparison with transgalactosylation under MWI at 300 W including a 5 times higher enzyme activity (60 U/mL) (Figure 5).

**Reaction Time (min)**	**TH 85 °C**				**MWI 300 W**			
	**GlcNAc-Linker-*t*Boc**		**Gal(β1,4)GlcNAc-Linker-*t*Boc**		**GlcNAc-Linker-*t*Boc**		**Gal(β1,4)GlcNAc-Linker-*t*Boc**	
	**rel. Peak Area (%)**	**STD**	**rel. Peak Area (%)**	**STD**	**rel. Peak Area (%)**	**STD**	**rel. Peak Area (%)**	**STD**
0	100.0	0.00	0.0	0.00	100.0	0.00	0.0	0.00
5	59.5	7.93	40.4	7.81	54.4	6.58	44.9	6.37
10	49.9	4.51	48.7	3.83	46.6	3.44	51.9	3.09
15	47.3	0.27	50.0	1.23	45.1	2.69	52.5	2.26
30	48.6	3.93	45.3	5.91	45.4	2.26	50.5	1.77
60	52.5	4.07	36.8	6.60	48.2	1.62	44.7	0.39
